# Barley *ABI5* (*Abscisic Acid INSENSITIVE 5*) Is Involved in Abscisic Acid-Dependent Drought Response

**DOI:** 10.3389/fpls.2020.01138

**Published:** 2020-07-29

**Authors:** Anna Collin, Agata Daszkowska-Golec, Marzena Kurowska, Iwona Szarejko

**Affiliations:** Institute of Biology, Biotechnology and Environmental Protection, Faculty of Natural Sciences, University of Silesia in Katowice, Katowice, Poland

**Keywords:** abscisic acid, monocots, barley, *Hordeum vulgare*, water deficit, stress, transcriptomics, ABI5

## Abstract

ABA INSENSITIVE 5 (ABI5) is a basic leucine zipper (bZIP) transcription factor which acts in the abscisic acid (ABA) network and is activated in response to abiotic stresses. However, the precise role of barley (*Hordeum vulgare*) ABI5 in ABA signaling and its function under stress remains elusive. Here, we show that *HvABI5* is involved in ABA-dependent regulation of barley response to drought stress. We identified barley TILLING mutants carrying different alleles in the *HvABI5* gene and we studied in detail the physiological and molecular response to drought and ABA for one of them. The *hvabi5.d* mutant, carrying G1751A transition, was insensitive to ABA during seed germination, yet it showed the ability to store more water than its parent cv. “Sebastian” (WT) in response to drought stress. The drought-tolerant phenotype of *hvabi5.d* was associated with better membrane protection, higher flavonoid content, and faster stomatal closure in the mutant under stress compared to the WT. The microarray transcriptome analysis revealed up-regulation of genes associated with cell protection mechanisms in the mutant. Furthermore, HvABI5 target genes: *HVA1* and *HVA22* showed higher activity after drought, which may imply better adaptation of *hvabi5.d* to stress. On the other hand, chlorophyll content in *hvabi5.d* was lower than in WT, which was associated with decreased photosynthesis efficiency observed in the mutant after drought treatment. To verify that *HvABI5* acts in the ABA-dependent manner we analyzed expression of selected genes related to ABA pathway in *hvabi5.d* and its WT parent after drought and ABA treatments. The expression of key genes involved in ABA metabolism and signaling differed in the mutant and the WT under stress. Drought-induced increase of expression of *HvNCED1, HvBG8, HvSnRK2.1*, and *HvPP2C4* genes was 2–20 times higher in *hvabi5.d* compared to “Sebastian”. We also observed a faster stomatal closure in *hvabi5.d* and much higher induction of *HvNCED1* and *HvSnRK2.1* genes after ABA treatment. Together, these findings demonstrate that *HvABI5* plays a role in regulation of drought response in barley and suggest that HvABI5 might be engaged in the fine tuning of ABA signaling by a feedback regulation between biosynthetic and signaling events. In addition, they point to different mechanisms of *HvABI5* action in regulating drought response and seed germination in barley.

## Introduction

Abscisic acid (ABA) is the crucial regulator of plant responses to abiotic stresses. In the presence of unfavorable conditions, the precise regulation and function of ABA-dependent signaling components ensure the appropriate activity of stress-responsive genes (reviewed by Yoshida et al., 2019), and thus the regulation of physiological processes, such as photosynthesis, stomatal closure (Song et al., 2016; Cai et al., 2017; Saito and Uozumi, 2019), and osmoprotectant biosynthesis (Jones, 2016; Sah et al., 2016; Martignago et al., 2020).

In *Arabidopsis thaliana*, *ABA INSENSITIVE 5* (*ABI5*) encodes the ABA-dependent, BASIC LEUCINE ZIPPER (bZIP) transcription factor, composed of C1, C2, C3, and bZIP conserved domains (Lopez-Molina et al., 2001; Nakamura et al., 2001). The bZIP domain is responsible for DNA binding, whereas C1, C2, and C3 domains are recognized and phosphorylated by protein kinases (Furihata et al., 2006). C3 is also crucial for ABI5 interaction with another transcription factor, ABI3 (Tezuka et al., 2013). AtABI5 plays a role during early ABA signaling and was shown to be activated shortly after the perception of a stress signal. The formation of ABA-PYRABACTIN RESISTANCE1/PYR LIKE/REGULATORY COMPONENT OF ABA RECEPTOR-PHOSPHATASE 2C complex (ABA-PYR1/PYL/RCAR-PP2C) promotes ABI5 phosphorylation mediated by the SNF1-RELATED PROTEIN KINASE2s (SnRK2s) and thus its activation. Then, AtABI5 binds ABA RESPONSIVE ELEMENTs (ABRE *cis*-elements) present in the promoters of regulated genes and activates or represses their transcription, often in the interaction with other regulatory proteins (reviewed by Daszkowska-Golec, 2016; Sah et al., 2016; Dejonghe et al., 2018; Yoshida et al., 2019).

*ABI5* was described as a regulator of seed germination and early seedling development in the presence of ABA and abiotic stresses (Finkelstein, 1994; Lopez-Molina et al., 2001; reviewed by Skubacz et al., 2016). Several Arabidopsis *abi5* alleles have been identified using insertional or physical mutagenesis (*Atabi5-1*, *Atabi5-2, Atabi5-4, Atabi5-5, Atabi5-7, Atabi5-8*, and *Atabi5-9*) (Finkelstein, 1994; Lopez-Molina and Chua, 2000; Carles et al., 2002; Nambara et al., 2002; Zheng et al., 2012; Tezuka et al., 2013). It was demonstrated that *Atabi5-2* and *Atabi5-5* were insensitive also to salt and osmotic stresses during seed germination (Carles et al., 2002), while *Atabi5-1* showed ABA, salt, and osmotic insensitivity both at germination and early seedling stage (Finkelstein et al., 2005; Yuan et al., 2011). Interestingly, the *Atabi5-1* showed no differences in other ABA-regulated processes, such as stomata closure during vegetative growth (Finkelstein, 1994; Finkelstein and Lynch, 2000). Although *Atabi5-1* was described as “not-wilty” by Finkelstein and Lynch (2000; http://www.arabidopsis.org, AT2G36270) no detailed analysis of the mutant under drought stress has been reported.

*AtABI5* expression was observed during a short developmental window, between 48 and 60 h after imbibition, in the presence of drought and salt stress. The increased activity of *AtABI5* was related to germination inhibition (Lopez-Molina et al., 2001; Maia et al., 2014). Additionally, *AtABI5* function was associated with the repression of primary and lateral root development (Lopez-Molina et al., 2001; Signora et al., 2001). It was proven that AtABI5 downstream target genes were responsible for the inhibition of germination, adaptation to reduced water availability, lower photosynthesis efficiency, and reactive oxygen species (ROS) scavenging (Finkelstein and Lynch, 2000; Kanai et al., 2010; Su et al., 2016; Bi et al., 2017).

Another level of abiotic stress response regulated by AtABI5 involves lipid metabolism where AtABI5 is able to activate the expression of gene encoding triacylglycerol biosynthesis enzyme (Kong et al., 2013). AtABI5 promotes chlorophyll catabolism and inhibits photosynthesis *via* transcriptional regulation of genes encoding a protein inducing destabilization of LIGHT-HARVESTING COMPLEX FOR PHOTOSYSTEM II (LHCPII) and chlorophyll *b* reductase (Sakuraba et al., 2014). AtABI5 also regulates expression of gene encoding detoxifying enzyme, *CATALASE1* (*CAT1*) (Bi et al., 2017). Recently, AtABI5 was described as a direct repressor of *PHOSPHATE1* (*PHO1*) gene associated with phosphate homeostasis (Huang et al., 2017).

AtABI5 undergoes complex regulation at the post-translational level by phosphorylation, ubiquitination, sumoylation, and nitrosylation. AtABI5 is phosphorylated by many kinases, such as SnRK2.2, SnRK2.3, CALCIUM-DEPENDENT PROTEIN KINASE 11 (CPK11), and SOS2-LIKE PROTEIN KINASE 5 (PKS5), which results in its activation (Lynch et al., 2012; Zhou et al., 2015). It is noteworthy that all types of AtABI5 modifications take place in the specific amino acid positions of the protein (Miura et al., 2009; Liu and Stone, 2013; Albertos et al., 2015; Yu et al., 2015).

In Arabidopsis AtABI5 is considered as the main regulator of ABA response only during seed germination and early seedling growth, whereas other bZIP transcription factors that recognize the ABRE *cis*-element, such as ABRE BINDING FACTOR2/ABRE-BINDING PROTEIN1 (AtABF2/AREB1), AtABF4/AREB2, AtABF3, and AtABF1 take part in ABA signaling in vegetative tissues under abiotic stresses (Fujita et al., 2005; Yoshida et al., 2015). It was shown that *Atabf4/areb2* and *Atabf3* mutants were more sensitive to drought than WT, whereas overexpression of *AtABF2/AREB1* ensured better drought tolerance due to increased expression of *LEA* genes (Fujita et al., 2005; Yoshida et al., 2010). AtABFs/AREBs promote stomatal closure and chlorophyll catabolism (Gao et al., 2016; Qian et al., 2019). Importantly, AtABF2/AREB1, AtABF4/AREB2, AtABF3, and AtABF1 show redundancy during regulation of ABA-mediated drought responses. *Atabf2/areb1 Atabf4/areb2 Atabf3 Atabf1* quadruple mutant showed 2.2% survival rate after drought treatment, whereas survival rate of single *Atabf/areb* mutants was 38.6–57.1% (Yoshida et al., 2010; Yoshida et al., 2015). However, sometimes *AtABF* genes can act autonomously, e.g. *AtABF3* regulates seedling root growth in the presence of ABA (Finkelstein et al., 2005; Yoshida et al., 2010). Moreover, the expression profile of *LEA* genes in the *Atabf2/areb1*, *Atabf4/areb2*, and *Atabf3* mutants is not always similar (Yoshida et al., 2010).

The function of barley (*Hordeum vulgare*) ABI5 homolog as an ABA-dependent transcription factor has been described first in barley seeds, mainly in aleurone layer by Casaretto and Ho (2003). The authors demonstrated that in the presence of ABA, HvABI5 binds ABRE cis-element present in ABA RESPONSE PROMOTER COMPLEX (ABRC). *HVA1* and *HVA22* encoding LEA proteins have been identified as the HvABI5-activated genes in barley aleurone cells (Casaretto and Ho, 2003). Both proteins ensure the protection from water deprivation in barley seeds. The expression of *HVA1* and *HVA22* is also dependent on VIVIPAROUS1 (HvVP1), an ortholog of AtABI3 (Casaretto and Ho, 2003). Probably, HvABI5 interaction with HvVP1 is required for HvABI5 activity, similarly to the interaction between ABI5 and ABI3 described in Arabidopsis (Casaretto and Ho, 2003; Bensmihen et al., 2004). Interestingly, *HvABI5* expression is auto-activated by HvABI5 protein, as it is observed for *AtABI5* in Arabidopsis (Finkelstein and Lynch, 2000; Casaretto and Ho, 2005). It was also found that HvABI5 activity as transcription factor is dependent on serine in 106 position, which probably serves as a target of ABA-dependent phosphorylation by *HvSnRK2.1* (*HvPKABA1*) (Casaretto and Ho, 2005).

HvABI5 shows high similarity to wheat wABI5, rice TRANSCRIPTION FACTOR RESPONSIBLE FOR ABA REGULATION1 (TRAB1), and maize ZmABI5. wABI5, TRAB1, and ZmABI5 were described as ABA-dependent transcription factors regulating abiotic stress responses (Kagaya et al., 2002; Kobayashi et al., 2008; Yan et al., 2012). wABI5 acts as a positive regulator of drought response *via* expression activation of LEA encoding genes: *DEHYDRIN13* (*wDHN13*), *RESPONSIVE TO ABA18* (*wRAB18*), and *wRAB19* (Kobayashi et al., 2008). The increased expression of *TRAB1* was observed in rice stress-tolerant cultivar under various abiotic stresses (Paul and Roychoudhury, 2019). On the other side, ZmABI5 negatively regulates abiotic stress responses. *ZmABI5* overexpression promoted chlorophyll degradation and reduced activity of detoxifying enzymes, peroxidase (POD), and superoxide dismutase (SOD), under abiotic stresses (Yan et al., 2012). Furthermore, other AtABI5 homologs were identified in rice (OsABI5) and in wheat (TaABI5). Similarly, to ZmABI5, OsABI5 was also described as a negative regulator of stress tolerance (Zou et al., 2008). Wheat *TaABI5* was shown to be active only in seeds. *TaABI5* overexpression in Arabidopsis seeds caused ABA-hypersensitive germination (Utsugi et al., 2020).

Given the fact that drought stress is considered as the cause of the most severe yield losses worldwide there is a need for extensive development of drought-tolerant cultivars. However, drought tolerance is a very complex trait showing quantitative inheritance and large genotype x environment interactions, therefore developing cultivars better adopted to water deficiency conditions remains a challenge for crop breeding programs (Agarwal et al., 2013; Gilliham et al., 2017). Identification and functional characterization of stress-related genes can help in obtaining plants with a higher tolerance to drought (Joshi et al., 2016; Bailey-Serres et al., 2019). Barley has been suggested as a cereal model for studying mechanisms of abiotic stress adaptation (Dawson et al., 2015), due to its natural ability to cope better with drought stress than other cereals. According to the Food and Agriculture Organization report, barley is the fourth most important cereal crop regarding the harvested acreage (FAO, 2018). These characteristics, together with the assembled genome sequence (IBSC_v2, Ensembl Plants), enhanced the role of barley as a model species for studying genetic and molecular processes underlying drought tolerance in cereals.

In this study we focused on elucidating the role of *HvABI5* in ABA signaling during barley response to drought stress. First, we have identified a *hvabi5.d* mutant using barley TILLING (Targeted Induced Local Lesions IN Genomes) platform created in our laboratory (Szurman-Zubrzycka et al., 2018). The identified TILLING mutant made it possible to initiate studies on the role of *HvABI5* in regulation of ABA and drought responses in barley. Taking advantage from Arabidopsis research on *abi5* mutants, we analyzed seed germination and early seedling of *hvabi5.d* in the presence of ABA. Next, we applied drought stress to *hvabi5.d* seedlings and measured a range of physiological parameters in order to describe the response of *hvabi5.d* to drought. To gain insight into molecular basis of *hvabi5.d* drought tolerance we conducted a global transcriptome analysis of the mutant and parent cultivar “Sebastian” exposed to drought treatment. Then, to answer the question whether *HvABI5* regulates drought response in the ABA-dependent manner, we analyzed the expression of selected genes related to ABA pathway in *hvabi5.d* and its wild type parent under drought and ABA treatments. We also studied stomatal conductance of analyzed genotypes after ABA treatment. Our results clearly indicated the role of *HvABI5* as the ABA-dependent regulator of response to drought stress in barley. In addition, these findings clearly pointed to different mechanisms of *HvABI5* action in regulating drought response and seed germination.

## Material and Methods

### Plant Material

The *hvabi5.d* mutant was identified using a TILLING platform (*Hor*TILLUS) developed after chemical mutagenesis of spring barley cultivar “Sebastian” in our laboratory (Szarejko et al., 2017; Szurman-Zubrzycka et al., 2018). The DNA samples from 6,144 M_2_ plants of the *Hor*TILLUS population were screened with the aim to identify mutations in *HvABI5* gene. TILLING of *HvABI5* gene was performed based on *HvABI5* sequence for “Sebastian” variety present in GenBank (GenBank acc. no. HQ456390.1). Conserved regions of *HvABI5* were mapped using CODDLE (Codons Optimised to Discover Deleterious Lesions; http://www.proweb.org/coddle/) and CLUSTAL OMEGA (http://www.ebi.ac.uk/Tools/msa/clustalo/) tools. The analyzed TILLING fragment (1,072 bp) embraced exons encoding most of C1, C2, C3, and bZIP domains, highly conserved and crucial for *HvABI5* function. The mutational screening was performed according to the procedure described by Szurman-Zubrzycka et al. (2018). Mutants carrying nucleotide substitutions in homozygous state were identified in M_2_ or M_3_ generation and for most of missense mutants, seeds were increased in M_4_-M_5_ generation. The type and state of mutation (homo/heterozygous) was analyzed using the CodonCode Aligner software. The *hvabi5.d* allele was identified in the heterozygous state, therefore homozygous plants were selected in the segregating M_3_ progeny before using *hvabi5.d* mutant in the studies. Seven mutants carrying different alleles: alleles (*hvabi5.b*, *hvabi5.d*, *hvabi5.e*, *hvabi5.i*, *hvabi5.o*, *hvabi5.u*, *hvabi5.w*) were used for preliminary relative water content (RWC) screening after drought treatment in M_4_-M_6_ generation. Mutant carrying the *hvabi5.d* allele was selected for a detailed physiological and molecular studies presented below. Additionally, the homozygous line of *hvabi5.d* mutant was backcrossed twice with parent variety “Sebastian” to clean the mutant genome from the possible background mutations. The obtained homozygous F_4_BC_2_ lines with *hvabi5.d* allele were further used for basic physiological tests after drought treatment and expression analysis of selected genes.

### Prediction of Mutation Significance Using Bioinformatics Software

The conservation of mutated position was checked at the protein level in Clustal Omega (http://www.ebi.ac.uk/Tools/msa/clustalo/) using multi sequence alignment of *ABI5* sequences in dicot and monocot species.

### Germination Assay in the Presence of ABA

Thirty seeds of *hvabi5.d* and wild-type (WT) parent cultivar “Sebastian” were sown in a Petri dish (ф=90 mm) containing two layers of Whatman filters with 5 ml distilled water (control) or ABA solution (75 and 300 μM). Four-day stratification at 4°C in darkness was applied to synchronize seed response. Then, the seeds were germinated in a growth chamber (22°C, 16-h-light/8-h darkness, 200 μmol/m^−2^ s^−1^ illumination). Germination process was evaluated by a visible appearance of a root on the 1^st^, 2^nd^, 3^rd^, and 4^th^ day after stratification. Germination assay was performed in three biological replications (each Petri dish containing 30 seeds was considered as one biological replicate) and the experiment was repeated three times.

### Seedling Development Assay in the Presence of ABA

Four-day-old seedlings of *hvabi5.d* and WT were used in this experiment. Plants were placed in glass tubes containing 90 ml of Murashige-Skoog (MS) medium or MS supplemented with 50 µM ABA. The treatment was applied for 6 d in a growth chamber (22°C, 16/8 h photoperiod, 200 μmol/m^−2^ s^−1^ illumination). The length of the first leaf and the longest seminal root were measured before and after the treatment. At the end of the treatment, chlorophyll *a* fluorescence was measured and the leaf tissue (0.02–0.05 g) was collected to extract proline. All analyzes were performed in three biological replications, with two plants per replication. The experiment and material collection were repeated three times.

#### Chlorophyll a Fluorescence

Chlorophyll *a* fluorescence was measured with the PocketPea fluorimeter (Hansatech Instruments Ltd., England). Before measurement, the leaves were adapted for 30 min in darkness. Next, the leaves were exposed to a pulse of saturating light [3,500 μmol (photon) m^−2^ s^−1^] for 1 sec. To analyze the chlorophyll *a* fluorescence, the JIP-test was applied (Strasser et al., 2004; Kalaji et al., 2016). The OJIP fluorescence transients consist of four phases: O-initial fluorescence level, J-fluorescence at 2 ms, I- fluorescence at 30 ms, and P-maximal fluorescence. The OJIP transients were used to calculate the following parameters: PI_ABS_—performance index for the photochemical activity and φP_0_—maximum quantum yield of primary photochemistry (Strasser et al., 2000).

#### Proline Content

Proline concentration (µmol g^−1^ fresh weight) was determined according to the colorimetric method of Carillo and Gibbon (2011). The absorbance was read at 520 nm using Victor X5 Multilabel Reader (PerkinElmer). The proline content was calculated according to the formula:

$$\text{Proline in μmol}/\text{g FW}=\frac{\text{Ab}{\text{s}}_{\text{extract}}-\text{blank}}{\text{slope}}\times \frac{\text{Vo}{\text{l}}_{\text{extract}}}{\text{Vo}{\text{l}}_{\text{aliquot}}}\times \frac{1}{\text{FW}}$$

where: Abs_extract_ is the absorbance of plant extract, blank is the absorbance of clear extraction solution, slope is determined by linear regression of a calibration curve, Vol_extract_ is the total extract volume, Vol_aliquot_ is the extract volume used for the assay, FW is the weight of the plant material.

### Drought Stress Experiment

#### Drought Stress Treatment

Drought stress was applied as described earlier (Daszkowska-Golec et al., 2017). Briefly, the experiment was carried in boxes (400 x 140 x 175 mm) filled with soil containing a mixture of sandy loam and sand (7:2) with known physicochemical properties. The soil was supplied with nutrient medium (**Supplementary Material S1**). Based on performed calculations, the water was easily available for plants at 14% of volumetric water content (vwc) in the soil, whereas the severe drought stress was achieved at 1.5%. When plants were grown in 14% vwc, the RWC in leaves was approximately 100%. The soil moisture was measured every day using time-domain reflectometer (TDR) EasyTest (Institute of Agrophysics, Polish Academy of Sciences, Poland). The WT and mutant plants were grown in a greenhouse for 10 d after sowing (DAS) under optimal water conditions (14% vwc), 20/18°C day/night, with a 16/8 h photoperiod, and 420 μE m^−2^ s^−1^ light intensity which was provided by fluorescent lamps. Afterward, the soil moisture was decreased by withholding the irrigation under the control of TDR measurements. On 15 DAS, when the soil moisture decreased to 3%, the plants were moved into a growth chamber, where temperature regime was set to 25°C/20°C day/night, with a 16/8 h photoperiod and 420 μEm^−2^ s^−1^ light intensity. The severe drought stress (1.5% vwc) lasted 10 d (16–25 DAS). The control plants were grown under the same conditions with optimal water supply (14% vwc) in parallel to the drought treated plants. On 25 DAS the RWC was evaluated for each genotype, according to the procedure described below.

The second leaves were collected from *hvabi5.d* and WT plants before water withdrawal (10 DAS) and after severe drought (25 DAS) and used for RNA extraction. The anthocyanins content index, flavonols content index, chlorophyll *a* fluorescence, and chlorophyll content index were analyzed before water withdrawal (10 DAS) and after drought stress (25 DAS). The stomatal conductance was measured under optimal water conditions (10 DAS), on the drought onset (13 and 15 DAS) and after severe drought (25 DAS). On the 25 DAS, RWC, electrolyte leakage (EL), and ABA content were analyzed using plants growing under optimal water supply and under drought stress. The schedule of drought experiment with indicated time-points of all assays are presented in **Supplementary Material S2**. We conducted all analyses using the second leaf since it was already present when plants entered drought treatment. Each genotype was tested in three biological replicates. One box containing 15 plants per genotype was considered as one replicate.

#### Relative Water Content

RWC was measured in the second leaf on the last day of drought stress (25 DAS). RWC parameter was calculated according to formula:

$$\text{RWC}=\frac{{\text{F}}_{\text{w}}-{\text{D}}_{\text{w}}}{{\text{T}}_{\text{w}}-{\text{D}}_{\text{w}}}\times 100\%$$

where: F_w_ (fresh weight) is the weight of detached leaf, T_w_ (turgid weight) is the weight of a leaf after 24 h rehydration in distilled water (leaves were submerged in distilled water in darkness), and D_w_ (dry weight) is the weight of a leaf dried at 60°C for 48 h. The measurement of RWC was performed in three biological replications (each biological replicate included leaves of three independent seedlings).

#### Electrolyte Leakage Analysis

The EL was analyzed in drought-treated and control seedlings on 25 DAS (according to the protocol of Bandurska and Gniazdowska-Skoczek, 1995). Briefly, the fragments of the middle part of the second leaf were washed three times in deionized water and kept at 10°C for 24 h. Afterward, the samples were transferred to the room temperature and EL was measured using pH/conductivity meter (CPC-505, Elmetron, Poland). Then, samples were autoclaved for 15 min to damage cells completely and conductivity was measured again at room temperature. Three replications were made for each treatment combination (each biological replicate included leaves of three seedlings). EL was calculated according to formula:

EL  (%  of  initial  measurement)=[1−(1−D1/D2)/(1−C1/C2)]×100

Where: D1 and C1 are the first conductivity measurements for respectively drought and control samples, whereas D2 and C2 are the second (after auctoclaving) conductivity measurements.

#### Flavonol, Anthocyanin, and Chlorophyll Content Indices Measurement

The measurements of flavonol, anthocyanin, and chlorophyll content indices were performed on the second leave on the 10 and 25 DAS using a Dualex Scientific+TM (Force-A, France). The measurements were performed in three biological replicates, with four seedlings per replicate.

#### Stomatal Conductance

Stomatal Conductance (mmol m^−2^ s^−1^) was measured using AP4 porometer (DELTA-T Devices, Burwell, UK). Measurements were conducted in three biological replicates, each replicate contained three individual plants. The analysis was performed at the exposed, central part of the second leaf adaxial side on 10, 13, 15, and 25 DAS, in three biological replications (each biological replicate included leaves of three independent seedlings).

#### Endogenous ABA Content

ABA content was measured according to the protocol of Nakurte et al. (2012). A modular HPLC system (Shimadzu, Japan) with SPD-M20A photodiode array detector and a Kinetex™ C18 (4.6 × 250 mm, 5 µm) column (Phenomenex) were used to conduct chromatography. The injection volume was 20 µl and the analysis was performed in the isocratic mode at a flow rate of 1 ml min^−1^. LabSolutions software was used to evaluate the results (Shimadzu, Japan).

### RNA Isolation

RNA was extracted from the second leaves (50–100 mg) of *hvabi5.d* and WT plants collected on 10 and 25 DAS in three biological replications (each biological replicate represented leaf of one seedling). RNA isolation was conducted using TriPure reagent, according to the modified Chomczynski’s method (Chomczyński and Sacchi, 1987). For the microarray analyses, RNA was additionally purified using precipitation in 1 M lithium chloride and each RNA precipitate was then dissolved in 15 μl of nuclease-free H_2_O. NanoDrop (ND-1000) spectrophotometer (NanoDrop Technologies, Wilmington, USA) was used for concentration quantification and quality check. RNA integrity was analyzed using Agilent 2100 Bioanalyzer with RNA 6000 Nano chip (Agilent Technologies, Santa Clara, USA).

### Microarray Analysis

#### Microarray Data Analysis

The synthesis, labeling, and hybridization of cDNA and cRNA were performed by the Genomics Core Facility, European Molecular Biology Laboratory (EMBL), Heidelberg, Germany. The microarray data were analyzed using GeneSpring GX 13.0 software (Agilent Technologies) as described earlier (Daszkowska-Golec et al., 2017). A gene was considered to be differentially expressed when the level of its expression differed between the analyzed conditions by at least two times (fold change (FC) ≥ 2; P ≤ 0.05 after FDR correction). The annotation of the Agilent Barley Gene Expression Microarray (Agilent Technologies) was performed against IBSC_v2 of barley genome deposited in Ensembl Plants (v. 45). Functional annotation of differentially regulated genes was carried out using Ensembl Plants tools and the IPK Barley BLAST Server as references (https://plants.ensembl.org/index.html, https://webblast.ipk-gatersleben.de/barley_ibsc/). Three biological replications were used for microarray expression analysis (each biological replicate represented leaf of one seedling).

#### GO Enrichment Analysis

The GO enrichment analysis was performed using AgriGO 2.0 (http://systemsbiology.cau.edu.cn/agriGOv2/) to identify the functional ontologies for differentially expressed genes (DEGs) and to obtain the categories of biological functions which were overrepresented in the analyzed groups of DEGs. The AgriGO 2.0 enrichment tool reveals the GO terms in the analyzed set of genes in comparison to the GO frequency in the background genome. The statistical significance of identified GO terms was assessed by the hypergeometric distribution followed by the Bonferroni method for multiple testing correction (corrected P *≤* 0.01).

### RT-qPCR Analysis of ABA-Related Genes

cDNA was synthetized using 1 µg of total RNA and ReverseAid First Strand cDNA Synthesis Kit (Thermo Scientific). The obtained volume of cDNA (20 µl) was diluted using water (1:5) in order to prepare template for RT-qPCR reaction. Primers were designed using Quant-Prime software (http://www.quantprime.de) and their sequences are given in **Supplementary Material S3**. The RT-qPCR was performed using SYBR GREEN I on LightCycler 480 Real-Time PCR Instrument (Roche). The program consisted of the initial denaturation step, followed by 45 cycles of 10 s at 95°C, 20 s at 60°C, and 10 s at 72°C. The LinReq software (Ramakers et al., 2003) was used for data analysis. Each sample was normalized using *ELONGATION FACTOR 1-α* (*EF1*) and *GLYCERALDEHYDE-3-PHOSPHATE DEHYDROGENASE* (*GAPDH*) reference genes (Rapacz et al., 2012). The relative expression level was calculated using expression of reference gene and gene of interest in WT under control conditions, according to delta-delta C_t_ method (Livak and Schmittgen, 2001). Three biological replications were used for gene expression analysis, each sample was analyzed in two technical replicates.

### Prediction of Promoter Transcription Factor Binding Sites

Analysis of promoters of selected ABA pathway genes was conducted using PlanPan 3.0 (http://plantpan.itps.ncku.edu.tw/promoter.php) and PlantRegMap (http://plantregmap.cbi.pku.edu.cn/) platforms. Analyzed promoter sequences included 1,000 bp before the START codon.

### ABA Spray Assay

Ten-day-old WT and *hvabi5.d* seedlings grown in growth chamber (20°C, 16/8 h photoperiod, 200 μmol/m^−2^ s^−1^ illumination) under optimal water conditions were sprayed with 25 ml distilled water or 200 μM ABA dissolved in distilled water according to the procedure proposed by Al-Momany and Abu-Romman (2014). The stomatal conductance (mmol m^−2^ s^−1^) was measured after 30 min, 3, and 6 h using AP4 porometer (DELTA-T Devices, Burwell, UK). The second leaves were collected from *hvabi5.d* and WT plants at each time-point and used for RNA extraction. The experiment was performed in five biological replications (each biological replicate represented leaf of one seedling). The method of expression analysis of selected ABA-related genes is described in *RT-qPCR Analysis of ABA-Related Genes*.

## Results

### Sequence Analysis of HvABI5 and Its Homologues

**Figure 1:**
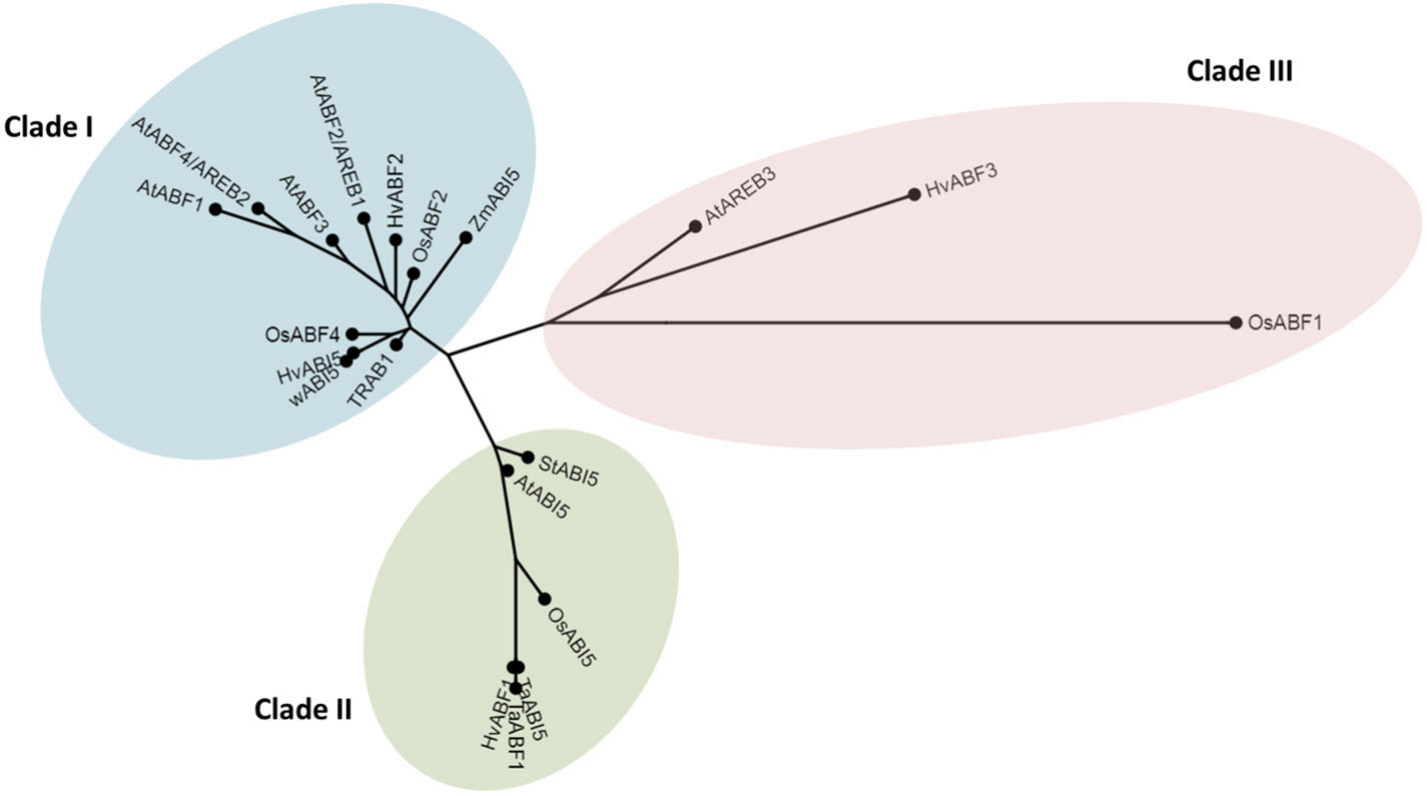
Relationships between HvABI5 and its homologs. Phylogenetic tree was generated using Phylogeny.fr software (http://www.phylogeny.fr/).

We analyzed the genetic distance between HvABI5 (HORVU5Hr1G068230) and its Arabidopsis, rice (*Oryza sativa*), wheat (*Triticum aestivum*), and maize (*Zea mays*) homologs. Based on phylogenetic analysis, bZIP transcription factors closely related to HvABI5 grouped in three clades (**Figure 1**). HvABI5 amino acid sequence formed a clade together with most of AtABFs/AtAREBs in Arabidopsis, HvABF2 (HORVU7Hr1G035500) in barley, OsABF2 (Os06t0211200), OsABF4 (Os09g0456200) and TRAB1 (Os08t0472000) in rice, wABI5 (TraesCS5A02G237200) in wheat, and ZmABI5 (Zm00001d018178) in maize. AtABI5 grouped in another clade, together with barley HvABF1 (HORVU3Hr1G084360). HvABI5 showed the highest level of amino acid sequence similarity to wABI5 (94.3%), OsABF4 (73.2%), TRAB1 (62.4%), OsABF2 (56.7%), and ZmABI5 (56.4%) (**Supplementary Material S4**). Among Arabidopsis genes, the most similar to HvABI5 were AtABF4/AREB2 (52.7%), AtABF2/AREB4 (51.3%), and AtABF3 (50.2%), however AtABI5 also showed a high percentage of similarity to HvABI5 (41.6%). Therefore, we could not clearly indicate the ortholog of HvABI5 in Arabidopsis. Despite the higher similarity of HvABI5 to AtABF/AtAREBs than to AtABI5, we decided not to change its name, since this gene (HORVU5Hr1G068230, Ensembl Plants) was described as HvABI5 in the previous studies of Casaretto and Ho (2003); Seiler et al. (2014) and Ishibashi et al. (2017). Furthermore, our results obtained during physiological analyses of barley *hvabi5.d* mutant pointed to its clear and obvious ABA-dependent and drought-tolerant phenotype, which was not the case of the single *abf* mutants in Arabidopsis. Taking these findings together, we decided to use the HvABI5 as a name of an ABA-dependent drought regulator described in this study.

### Initial Screening of *hvabi5* Mutants for Drought Stress Response

**Figure 2:**
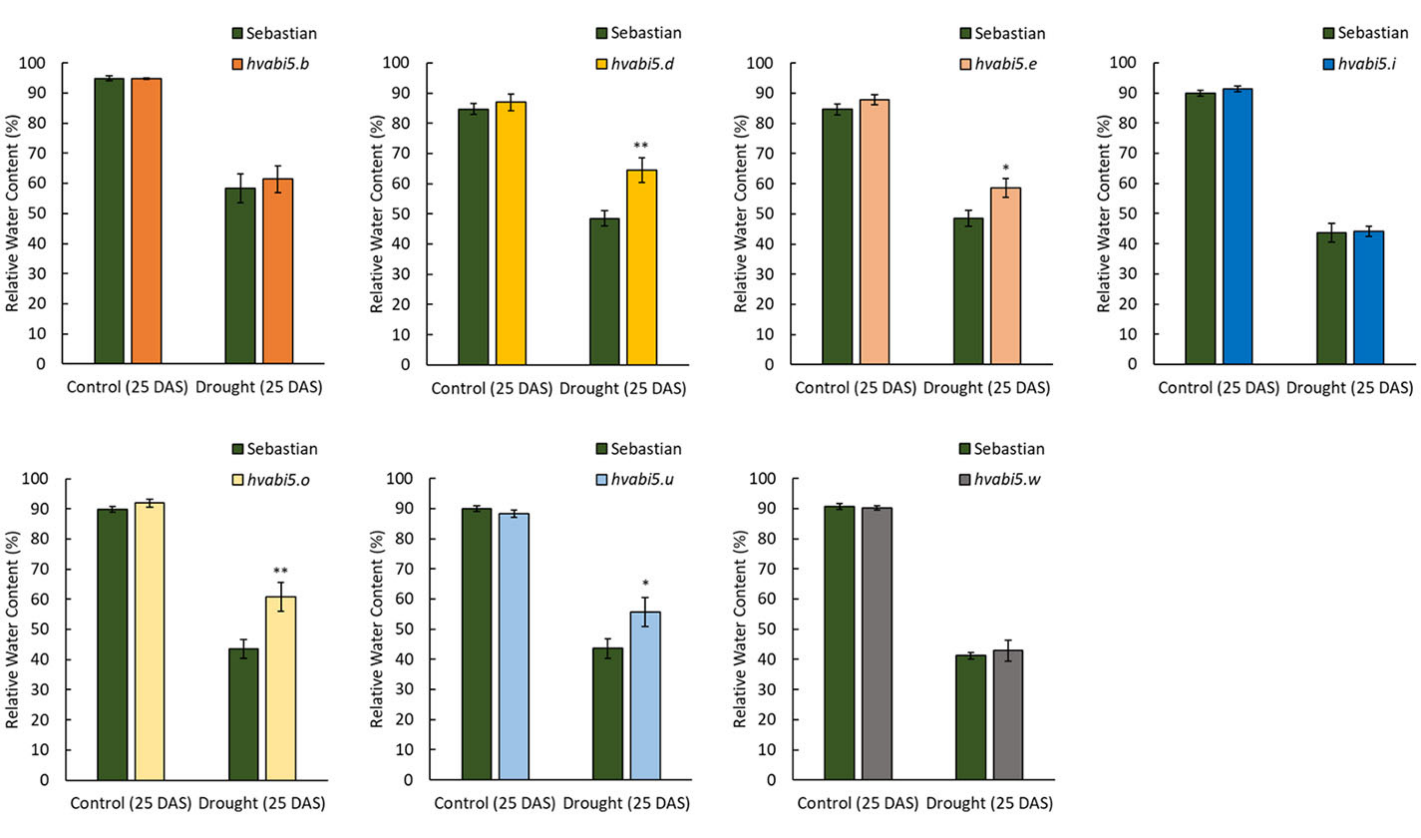
The relative water content on 25 DAS under control conditions and 10-d drought treatment in *hvabi5.b*, *hvabi5.d, hvabi5.e, hvabi5.i, hvabi5.o, hvabi5.u, hvabi5.w* mutants and parent cv. “Sebastian”. The statistical analysis was performed using the one-way ANOVA followed by Fisher’s least significant difference test (LSD-test) to assess the differences between analyzed genotypes—*P ≤ 0.05, **P ≤ 0.01.

We identified 14 missense mutations in *HvABI5* gene (Ensembl Plants, Gene ID HORVU5Hr1G068230; GenBank acc. no. HQ456390.1) after screening of 6,144 M_2_ plants of *Hor*TILLUS population. All identified mutations were confirmed by sequencing and most of them were G/C to A/T transitions. Seven of 14 identified mutants (**Table 1**, **Supplementary Material S5**), after seed increase in M_3_/M_4_ generation, were used for RWC screening after drought treatment. Four of the tested mutants (*hvabi5.d*, *hvabi5.e*, *hvabi5.o*, *hvabi5.u*) exhibited higher RWC than cv. “Sebastian” after exposure to 10-d of severe drought stress (1.5% vwc), while three other (*hvabi5.b*, *hvabi5.i*, *hvabi5.w*) showed no differences in RWC value compared to “Sebastian” (**Figure 2**). Based on RWC screening after drought treatment, conservation of positions of analyzed mutations in HvABI5 protein (**Supplementary Material S5**) and seed availability, we selected *hvabi5.d* mutant for further analysis.

**Table 1:** Mutants carrying missense mutations in *HvABI5* gene (Ensembl Plants, Gene ID HORVU5Hr1G068230; GenBank acc. no. HQ456390.1) that were analyzed in initial drought stress experiment using Relative Water Content (RWC) test.

Allele	Mutation position in *HvABI5* gene	Position of amino acid substitution in HvABI5 protein	Region in HvABI5 protein	State of mutation in M_2_ plant
*hvabi5.b*	G1621A	A231T	Non conserved	Homozygous
*hvabi5.d*	G1751A	R274K	Close to bZIP domain	Heterozygous
*hvabi5.e*	G1588A	D220N	Non conserved	Homozygous
*hvabi5.i*	G1229A	G100D	Close to C2 domain	Homozygous
*hvabi5.o*	T1135G	F69V	C1 domain	Homozygous
*hvabi5.u*	G1346A	G139E	Close to C2 domain	Homozygous
*hvabi5.w*	C1445T	P172L	Close to C3 domain	Homozygous

**Figure 3:**
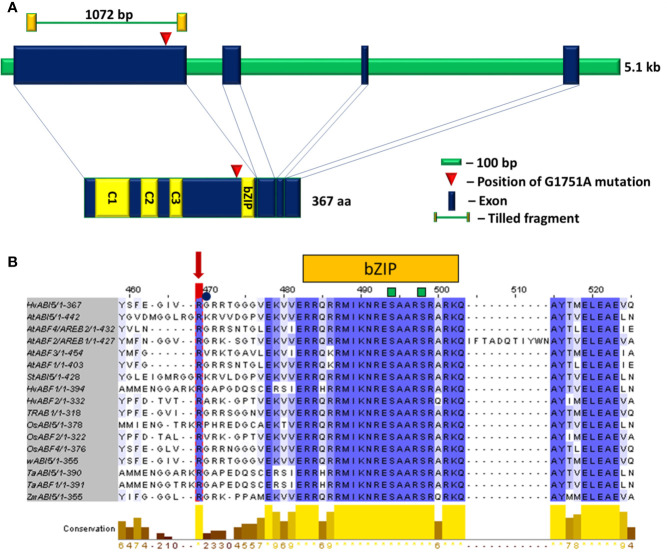
**(A)** The structure of *HvABI5* gene and HvABI5 protein with the position of *hvabi5.d* mutation indicated. C1, C2, C3—conserved charged domains, bZIP—basic leucine zipper domain. **(B)** An alignment of ABI5 and ABF proteins fragment comprising *hvabi5.d* mutation position. The changed arginine (R274) in *hvabi5.d* is marked by a red arrow. The square and circle mark phosphorylation and ubiquitination sites, respectively. Blue color indicates conservation of aligned position, yellow bars mark level of conservation. The numbering visible in alignment visualization refers to multi sequence alignment of 17 ABI5 and ABF protein sequences in dicot and monocot species. It does not indicate the position of amino acids substituted in the *hvabi5.d* mutant given in Tab.1.

*hvabi5.d* carries a G1751A transition (position according to Ensembl Plants, Gene ID HORVU5Hr1G068230) which results in the arginine to lysine substitution (R274K) located close to the bZIP domain (**Figure 3A**), at the highly conserved position across monocot and dicot species (**Figure 3B**; alignment of the entire ABI5 and ABF protein sequences is presented in **Supplementary Material S5**). Moreover, the *hvabi5.d* mutation is located nearby the ubiquitination site (K344) of AtABI5 described in Arabidopsis (Liu and Stone, 2013).

### Study of *hvabi5.d* Sensitivity to ABA at Seed Germination and Seedling Development Stages

**Figure 4:**
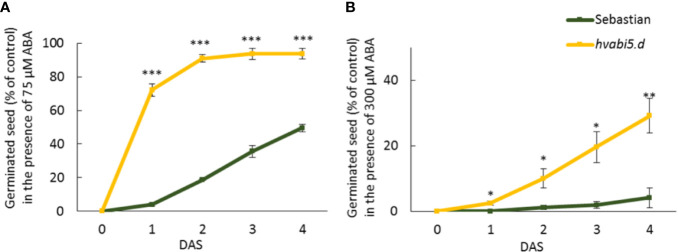
Germination of *hvabi5.d* and WT “Sebastian” under 75 **(A)** and 300 µM ABA **(B)**. DAS—day after stratification. The seed germination in the presence of ABA is presented as the percent of the control (untreated seeds) value. The statistical analysis was performed using the T-test to assess the differences between genotypes in the presence of ABA. Statistically significant differences are indicated by asterisks (*P ≤ 0.05. **P ≤ 0.01. ***P ≤ 0.001).

Taking advantage from *Atabi5* mutants, we addressed the question of *hvabi5.d* response to ABA during seed germination. The dynamics of germination of *hvabi5.d* and its WT “Sebastian” showed no difference under control conditions. However, after ABA treatment, *hvabi5.d* mutant turned out to be ABA-insensitive. The application of 75 µM ABA reduced germination of the WT to 50% of control, whereas no inhibition was observed in *hvabi5.d* (**Figure 4A**). Moreover, in the presence of 300 µM ABA, the rate of *hvabi5.d* germination reached approximately 30% of control, while germination of the WT seeds was almost completely inhibited (**Figure 4B**).

**Figure 5:**
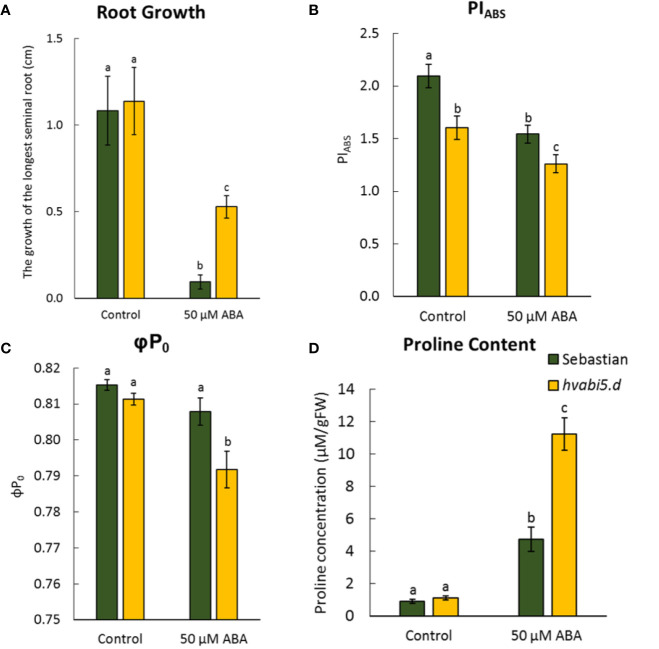
*hvabi5.d* response to ABA at seedling stage. **(A)** The growth of the longest seminal root of seedlings of analyzed genotypes after 6 d of ABA treatment, **(B)** photosynthesis performance index (PI_ABS_), **(C)** maximum quantum yield of primary photochemistry (φP_0_), and **(D)** proline accumulation in seedlings of analyzed genotypes under control conditions and ABA (50 µM) treatment. The statistical analysis was performed using the two-way ANOVA (P ≤ 0.05) followed by Tukey’s honestly significant difference test (Tukey HSD-test) (P ≤ 0.05) to assess the differences between different growth conditions and genotypes. Statistically significant differences (P ≤ 0.05) are marked by different letters.

Given the fact that *hvabi5.d* displayed ABA-insensitivity at germination stage, we analyzed early seedling development of *hvabi5.d* mutant and the WT parent in the presence of ABA. Although ABA negatively impacted the first leaf growth in both, WT and *hvabi5.d*, no significant differences in leaf length were observed between these genotypes either in control or exposure to ABA (**Supplementary Material S6**). However, *hvabi5.d* showed a much lower ABA-dependent root inhibition, compared to the WT (**Figure 5A**).

Taking into account the regulatory role of ABA in photosynthesis, we analyzed the efficiency of photosynthesis process in *hvabi5.d* and WT seedlings after ABA treatment. To investigate the photosynthesis performance, we applied the JIP test (Strasser et al., 2004). The obtained OJIP transients were converted into the biophysical parameters. The *hvabi5.d* seedlings grown under control conditions already showed a lower PI_ABS_ value than the WT. ABA treatment decreased the value of PI_ABS_ in both genotypes studied, nonetheless, *hvabi5.d* displayed a significantly more diminished PI_ABS_ value, compared to “Sebastian” (**Figure 5B**). The other analyzed parameter, φP_0_, was also lower in the mutant than in the parent cultivar after ABA treatment (**Figure 5C**). The values of φP_0_ and PI_ABS_ indicated the significant decrease of photosynthesis efficiency in *hvabi5.d* compared to “Sebastian”.

Apart from photosynthesis, we also measured the proline content in both genotypes. Proline is considered as an osmolyte that enables the osmotic adjustment during adaptation to abiotic stress. Although the proline content increased in response to ABA in both genotypes studied, the *hvabi5.d* accumulated twofold more proline than the WT (**Figure 5D**).

### *hvabi5.d* Exhibited Drought Tolerance

**Figure 6:**
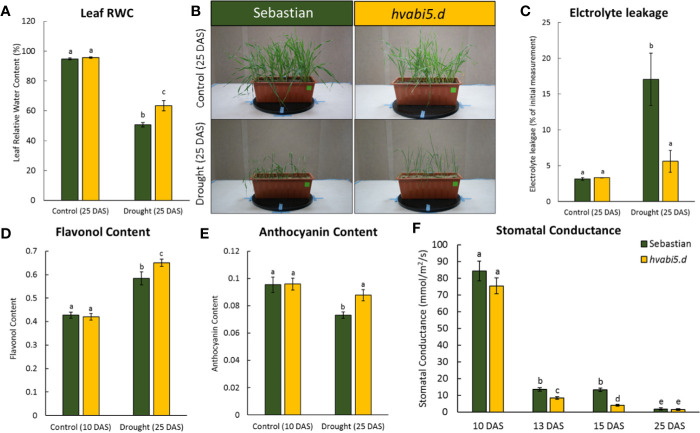
*hvabi5.d* response to drought stress. **(A)** The relative water content on 25 DAS under control conditions and drought in *hvabi5.d* and “Sebastian”. **(B)** The seedling of *hvabi5.d* and “Sebastian” on 25 DAS under control conditions and drought. **(C)** The EL on 25 DAS under control conditions and drought in *hvabi5.d* and “Sebastian” **(D)** The flavonol content **(E)** and anthocyanin content on the 10 and 25 DAS in *hvabi5.d* and “Sebastian”. **(F)** The stomatal conductance (g_s_) on the 10, 13, 15, and 25 DAS of analyzed genotypes. The statistical analysis was performed using the two-way ANOVA (P ≤ 0.05) followed by Tukey’s honestly significant difference test (Tukey HSD-test) (P ≤ 0.05) to assess the differences between different growth conditions and genotypes. Statistically significant differences (P ≤ 0.05) are marked by different letters.

Taking into consideration that *hvabi5.d* exhibited the ABA-insensitive phenotype at germination and early seedling stages, we investigated its reaction to drought stress in order to determine if *HvABI5* regulates drought response in barley. With the aim to characterize the physiological response of *hvabi5.d*, the leaf RWC, EL, and stomatal conductance were measured. No differences in leaf RWC between *hvabi5.d* and WT were observed under optimal water supply (control conditions) on 25 DAS. However, after 10-d exposure to drought stress, on the same 25 DAS, the mutant maintained 13% more water in leaf tissues than “Sebastian” according to the RWC value (**Figure 6A**). Moreover, drought stress induced more severe wilting in “Sebastian” compared to *hvabi5.d* (**Figure 6B**). Drought treatment induced a 5.4-fold increase of EL value in “Sebastian”, whereas in *hvabi5.d* there was no significant difference between control and drought-treated plants on 25 DAS (**Figure 6C**). We also analyzed flavonol and anthocyanin content index before and after drought treatment in both genotypes. Flavonols and anthocyanins function as antioxidants which scavenge toxic reactive oxygen species (ROS), protecting cells from their negative impact and thus contributing to drought tolerance. Similar levels of both, flavonols and anthocyainins were observed in both genotypes under well-watered conditions (10 DAS), but their content was significantly higher in *hvabi5.d* than in “Sebastian” under drought (25 DAS) (**Figures 6D, E**). These results are consistent with leaf RWC measurements. In order to follow the stomata action during the drought stress, we measured the stomatal conductance (*g_s_*) that describes the rate of gas exchange and transpiration between leaf and air. We analyzed this parameter before the drought treatment (10 DAS), at the drought onset (13 and 15 DAS), and after the severe drought stress (25 DAS) (**Figure 6F**). Both genotypes exhibited a vast reduction in stomatal conductance after severe drought. There were no significant differences between mutant and the WT when *g_s_* was measured before stress application (10 DAS) and after prolonged drought treatment (25 DAS) (**Figure 6F**). However, at the drought onset, the *hvabi5.d* showed respectively 1.5 and threefold lower stomatal conductance on 13 and 15 DAS than the parent variety. The decreased stomatal conductance enables the reduced transpiration *via* stress-induced stomata closure. It can be assumed that the better response of *hvabi5.d* to drought may be associated with a faster stomata closure and therefore, the lower water loss.

**Figure 7:**
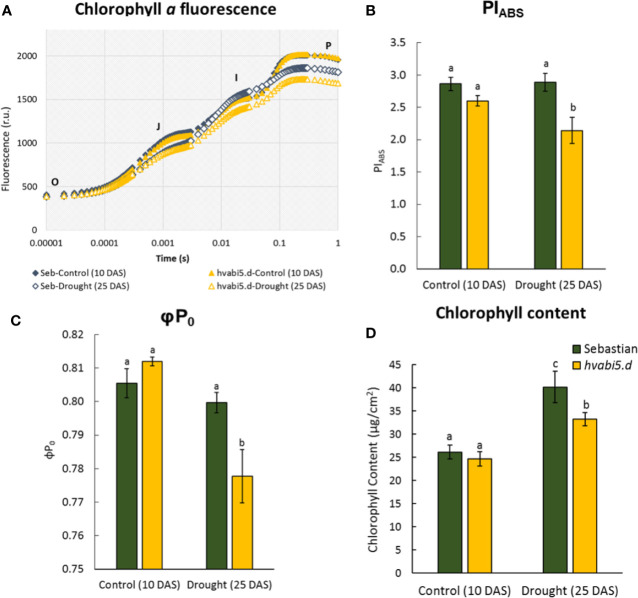
The effect of drought stress on photosynthesis efficiency in *hvabi5.d* and cv. “Sebastian”. **(A)** Chlorophyll *a* fluorescence curves during the pulse of light excitation, **(B)** performance index (PI_ABS_), **(C)** maximum quantum yield of primary photochemistry (φP_0_), and **(D)** chlorophyll content in analyzed genotypes under optimal water supply (10 DAS) and drought conditions (25 DAS). The statistical analysis was performed using the two-way ANOVA (P ≤ 0.05) followed by Tukey’s honestly significant difference test (Tukey HSD-test) (P ≤ 0.05) to assess the differences between different growth conditions and between genotypes. Statistically significant differences (P ≤ 0.05) are marked by different letters.

Taking into account that *hvabi5.d* showed the impaired photosynthesis in response to ABA at early seedling growth, we analyzed photosynthesis performance of *hvabi5.d* and WT when exposed to drought. First, the fluorescence of chlorophyll *a* was analyzed as the OJIP transient fluorescence (**Figure 7A**), where the J-I phase is related to reduction of plastoquinone (PQ) and cytochrome (Cyt b_6_f) and the I-P phase is associated with the reduction of the electron at the acceptor site of photosystem I (PSI) (Kalaji et al., 2016). The drought stress caused decrease of fluorescence only at the I-P phase in the WT, while in the *hvabi5.d* the decrease of the curve was observed already at the J-I phase and was even much more pronounced at the I-P phase (**Figure 7A**). These results indicate that photosynthesis is disturbed in *hvabi5.d* under drought. Moreover, some of the analyzed biophysical photosynthesis parameters were also impaired in *hvabi5.d* in the presence of drought (25 DAS), among them PI_ABS_ and φP_0_ (**Figures 7B, C**, **Supplementary Material S7**). PI_ABS_ indicates the amount of absorbed energy by electron acceptors, therefore its lower value denotes the impaired photosynthesis efficiency. The decreased photosynthesis efficiency in the mutant under drought might partially result from a more affected, compared to WT, stomatal conductance observed in the *hvabi5.d* at the onset of drought. It should be noted that under control conditions no differences in PI_ABS_ parameter were noted between the analyzed genotypes. The differences in PI_ABS_ value of the mutant observed under control conditions in two experiments (ABA and drought treatment) could arise from the developmental stage of analyzed plants and different experimental conditions, such as temperature and light intensity. Taking into account the impaired photosynthesis of *hvabi5.d*, we analyzed the content of chlorophyll in both genotypes. We detected no difference in chlorophyll content in genotypes analyzed under control conditions (10 DAS) (**Figure 7D**). However, we observed a decreased chlorophyll content in the mutant, compared to the WT under drought stress (25 DAS). It could be considered as another, besides the reduced stomatal conductance, cause of decreased photosynthesis efficiency in *hvabi5.d*.

### The Expression of *HvABI5*-Related Genes Is Up-Regulated in *hvabi5.d* Under Drought Stress

**Figure 8:**
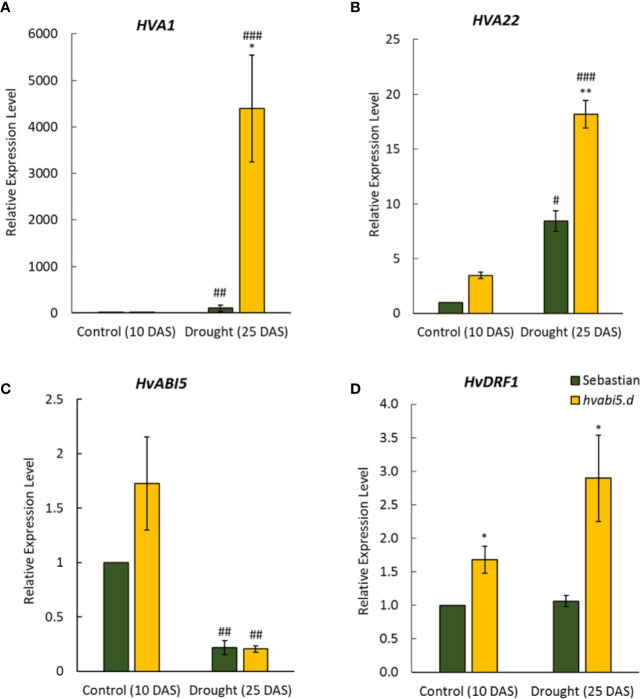
The expression of *HvABI5*-related genes in *hvabi5.d* and WT “Sebastian” under optimal water supply (10 DAS) and drought stress (25 DAS). *HvDRF1*—*DEHYDRATION‐RESPONSIVE FACTOR 1*. The statistical significance was estimated by T-test between analyzed genotypes—*P ≤ 0.05, **P ≤ 0.01 and between analyzed points—^#^P ≤ 0.05, ^##^P ≤ 0.01, ^###^P ≤ 0.001.

*hvabi5.d* showed response to ABA and drought tolerant phenotype, therefore we analyzed expression of *HvABI5*-related genes: *HVA1* and *HVA22* that are described as HvABI5 target genes (Casaretto and Ho, 2003). *HVA1* and *HVA22* expression was induced by drought stress in both genotypes, however it was, respectively, 43-fold and 2-fold higher in *hvabi5.d* than in the WT (**Figures 8A, B**). On the other side, drought induced a significant reduction of *HvABI5* expression, but we did not observed differences between both genotypes analyzed (**Figure 8C**). Therefore, we investigated expression of the gene encoding another transcription factor, DEHYDRATION-RESPONSIVE FACTOR 1 (HvDRF1), which is known to act synergistically with HvABI5 in activating *HVA1* (Xue and Loveridge, 2004). *HvDRF1* showed a significantly higher expression in the *hvabi5.d* mutant already under optimal water conditions compared to its WT (**Figure 8D**). Drought caused even more pronounced increase of *HvDRF1* expression in *hvabi5.d*, whereas no differences were observed in the WT.

### Transcriptome Analysis of *hvabi5.d* Under Drought Stress

Taking into account the drought tolerant phenotype of *hvabi5.d* and the higher expression of *HvABI5-*related genes, we anticipated that the global transcriptome analysis of this mutant under drought will help to shed light on the molecular mechanisms underlying its response to water deficit. For this purpose, we conducted the transcriptome analysis of *hvabi5.d* and its WT parent before drought treatment (10 DAS) and after 10-d drought stress application (25 DAS). The reliability of our microarray analysis was validated and confirmed using RT-qPCR analysis of genes which presented a wide range of expression levels in *hvabi5.d* under drought (**Supplementary Material S8**).

**Figure 9:**
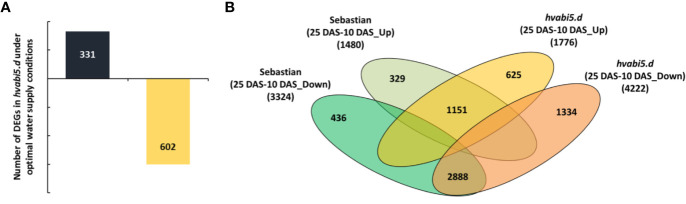
Transcriptome analysis of *hvabi5.d* and its WT parent “Sebastian” under optimal water conditions (10 DAS) and drought stress (25 DAS). **(A)** The number of differentially expressed genes (DEGs) in *hvabi5.d* under optimal water supply (10 DAS) (P ≤ 0.05 after FDR correction; FC ≥ 2). **(B)** Comparative analysis of the numbers of DEGs after drought in,Sebastian’ and the *hvabi5.d* mutant. In the Venn diagrams, the subsets of genes that were up-regulated or down-regulated specifically in “Sebastian” and in *hvabi5.d* after drought stress (25 DAS) compared to control conditions (10 DAS) are shown (P ≤ 0.05 after FDR correction; FC ≥ 2).

First, we addressed the question whether gene expression levels differed in *hvabi5.d* and “Sebastian” under optimal water supply, before the drought stress treatment. The comparison of *hvabi5.d* and WT transcriptomes revealed 933 DEGs (DEGs) in the mutant, among them 331 up- and 602 down-regulated genes (FC ≥ 2; P ≤ 0.05 after FDR correction) (**Figure 9A**). A relatively high number of DEGs could arise from the low cutoff criterion that was used in the analysis. Taking into account that *HvABI5* encodes a transcription factor, we decided to track expression level of a larger number of genes in order to avoid any oversight that might have happened if a stricter criterion was used for analysis. In order to get an insight into biological role of DEGs, the analysis of functional annotation was performed using AgriGO 2.0 (http://http://systemsbiology.cau.edu.cn/agriGOv2/).

Gene ontology (GO) analysis of the sets of genes specifically up- and down-regulated in the mutant indicated several over-represented biological processes (BP) associated with primary metabolism. In the set of up-regulated genes in *hvabi5.d* we identified processes such as: organonitrogen compound biosynthetic process, translation and protein folding (**Supplementary Material S9**). Furthermore, genes showing down-regulation specifically in *hvabi5.d* were categorized, among others, in BPs associated with ribonucleoside triphosphate and nucleoside triphosphate metabolic processes, ATP metabolic process and glycosyl compound metabolic process (**Supplementary Material S9**). The biological processes were considered as significant when P was less than 0.01 (assessed by the hypergeometric distribution followed by the Bonferroni method for multiple testing correction).

To investigate the transcriptome response to drought of *hvabi5.d* and its WT, we evaluated the gene expression level after drought stress (25 DAS) in relation to the conditions before drought treatment (10 DAS) for each genotype. Next, the DEGs were filtered out in order to obtain sets of genes specific for mutant and the WT. After drought treatment we found 625 up-regulated and 1,334 down-regulated genes specifically in *hvabi5.d*, whereas 329 and 436 genes were up- and down-regulated in “Sebastian”, respectively (FC ≥ 2; P ≤ 0.05 after FDR correction) (**Figure 9B**). To get an insight into the biological relevance of differentially regulated genes, we performed GO enrichment analysis of mutant- and “Sebastian”-specific sets of DEGs (**Supplementary Material S10**). It indicated that processes, such as: cellular amide metabolic process, translation, and gene expression were overrepresented within the set of genes specifically up-regulated in *hvabi5.d* under drought (**Supplementary Material S10**). Therefore, we carried out a more detailed analysis of this set of genes to reveal those, which could be associated with the drought tolerant phenotype of the *hvabi5.d* mutant (**Table 2**). Among them were genes directly involved in HvABI5 pathway, such as *HVA22* homolog *A* and *Ethylene-responsive transcription factor 4/dehydration responsive element binding protein 3* (*ERF4/DREB3*). Both these genes are engaged in barley response to drought and showed a significant increase of expression in our experiments (**Figure 8**). Furthermore, we identified other genes up-regulated specifically in *hvabi5.d*, which encode proteins associated with abiotic stress tolerance, such as late embryogenesis abundant proteins (LEAs), heat shock proteins (HSPs: Heat shock 70 kDa protein 3, Heat shock 70 kDa protein 15), and heat stress transcription factor C2b (HSFC2B). Some of them were also shown to be involved in detoxification of cytotoxic metabolites, among them genes encoding detoxifying enzymes: PRXs (peroxidase superfamily proteins), SOD ([Cu-Zn]2), and genes associated with flavonoid biosynthesis (*Anthocyanidin reductase*, *Flavanone 3-hydroxylase*, *Chalcone-flavonone isomerase*, and *MYB domain protein 30*). A closer look at the set of up-regulated genes in *hvabi5.d* revealed the phytohormonal crosstalk, since genes related to brassinosteroid (*Delta(24)-sterol reductase*), gibberelin (*Gibberellin receptor GID1*), and jasmonate (*Transcription factor MYC2*) signaling were identified in this subset (**Table 2**).

**Table 2:** Differentially expressed genes (DEGs) identified in *hvabi5.d* after drought treatment associated with adaptation to abiotic stress, flavonoid biosynthesis, detoxification, photosynthesis, and phytohormonal pathways.

Category	Gene ID	Fold change	Annotation
*Adaptation to abiotic stress*	HORVU7Hr1G012300	8.61	LEA
	HORVU6Hr1G081460	5.31	Heat shock 70 kDa protein 3
	HORVU7Hr1G038040	3.92	HVA22 homolog A
	HORVU1Hr1G060490	3.59	Ethylene-responsive transcription factor 4
	HORVU7Hr1G088920	2.98	Heat stress transcription factor C-2b
	HORVU1Hr1G027420	2.92	Heat shock 70 kDa protein 15
	HORVU5Hr1G120230	2.90	Late embryogenesis abundant protein
	HORVU1Hr1G059870	2.47	Late embryogenesis abundant protein D-19
*Flavonoid biosynthesis*	HORVU2Hr1G108250	33.04	Anthocyanidin reductase
	HORVU2Hr1G110130	6.19	Flavanone 3-hydroxylase
	HORVU5Hr1G046480	3.29	Chalcone-flavonone isomerase
	HORVU5Hr1G123880	2.17	Myb domain protein 30
*Detoxification*	HORVU4Hr1G057210	2.84	Peroxidase superfamily protein
	HORVU4Hr1G057170	2.84	Peroxidase superfamily protein
	HORVU2Hr1G021110	2.20	Superoxide dismutase [Cu-Zn] 2
*Photosynthesis*	HORVU7Hr1G096250	−8.18	High chlorophyll fluorescence phenotype 173
	HORVU6Hr1G033160	−3.53	Chlorophyll A/B binding protein 3
	HORVU3Hr1G021910	−3.35	Tetratricopeptide repeat (TPR)-containing protein
	HORVU5Hr1G109940	−2.22	Chaperonin-like RbcX protein
	HORVU5Hr1G093930	−2.22	Chaperonin-like RbcX protein
*Auxin pathway*	HORVU1Hr1G016700	−2.59	Auxin transporter-like protein 3
	HORVU7Hr1G084940	−2.53	Auxin-responsive protein IAA23
	HORVU3Hr1G032230	−2.28	Auxin response factor 1
	HORVU3Hr1G070620	−2.17	Auxin-responsive protein IAA6
	HORVU2Hr1G076920	−2.08	Auxin response factor 1
	HORVU3Hr1G097200	−2.05	Auxin response factor 2
*Gibberellin pathway*	HORVU4Hr1G062730	13.10	Gibberellin receptor GID1
	HORVU2Hr1G099440	−2.07	Terpene synthase 04
*Cytokinin pathway*	HORVU3Hr1G027460	−2.53	Cytokinin dehydrogenase 2
*Brassinosteroid pathway*	HORVU7Hr1G120030	4.18	Delta(24)-sterol reductase
	HORVU5Hr1G114850	−2.27	Protein kinase superfamily protein
	HORVU3Hr1G068020	−2.04	Leucine-rich receptor-like protein kinase family protein
*Jasmonic acid pathway*	HORVU1Hr1G050560	2.71	Transcription factor MYC2

Genes that were down-regulated specifically in *hvabi5.d* were found to be involved in biological processes related to primary metabolism e.g., translation, peptide metabolic process, organonitrogen compound biosynthetic process, cellular protein metabolic process, and gene expression (**Supplementary Material S10**). A detailed analysis of the down-regulated DEGs list in the mutant (**Table 2**) revealed genes associated with chlorophyll (*chlorophyll A/B binding protein 3* and *high chlorophyll fluorescence phenotype 173*), both related to photosynthesis efficiency. Other genes related to photosynthesis, such as genes encoding chaperones involved in RuBisCO assembly (chaperonin-like RbcX proteins) and chaperone associated with photosystem II assembly [tetratricopeptide repeat (TPR)-containing protein] were also identified in the down-regulated group (**Table 2**). Among the down-regulated set, several genes involved in auxin pathway (*auxin-responsive protein IAA6*, *auxin-responsive protein IAA23*, *auxin response factors 1*, *auxin response factor 2*, *auxin transporter-like protein 3*), brassinosteroid signaling (*leucine-rich receptor-like protein kinase family protein*, *Protein kinase superfamily protein*), gibberelin synthesis (*terpene synthase 04*), and cytokinin catabolism (*cytokinin dehydrogenase 2*) were identified (**Table 2**).

Based on transcriptome analysis of *hvabi5.d* response to drought, we assume that a better drought tolerance of the mutant results from of the higher activation of genes associated with the HvABI5 pathway and abiotic stress response mechanisms, together with the changed phytohormonal crosstalk. It should be underlined that we did not observe similar reactions to drought stress in the WT cv. “Sebastian” (**Supplementary Material S11**).

### The Expression of ABA-Related Genes Is Changed in *hvabi5.d* Under Drought Stress

Having shown that *hvabi5.d* mutant displayed ABA insensitivity during seed germination/early root growth and drought-tolerant phenotype at seedling/tillering stage, we asked next whether *HvABI5* regulates barley drought response through the ABA-dependent way. To answer this question, we first analyzed the drought stress response of the genes encoding ABA metabolism and ABA signaling in *hvabi5.d* and WT. We compared plants grown under optimal water supply before drought (10 DAS) and after drought stress (25 DAS).

**Figure 10:**
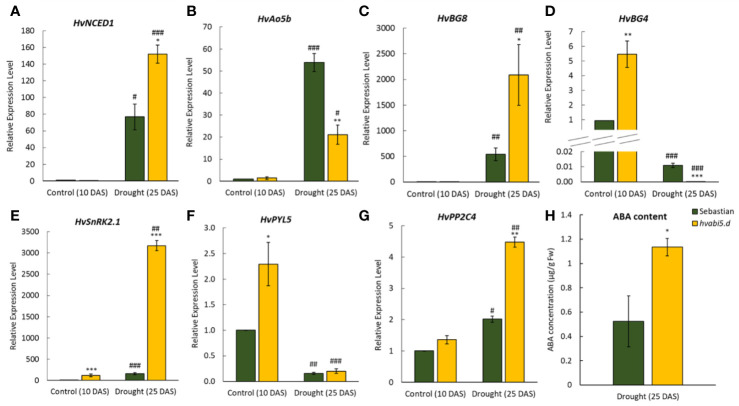
The expression of ABA pathway-related genes in *hvabi5.d* and its WT parent “Sebastian” under optimal water supply (10 DAS) and drought stress (25 DAS). **(A)** *HvNCED1*—*CAROTENOID CLEAVAGE DIOXYGENASE1*, **(B)** *HvAO5b*—*ABSCISIC ALDEHYDE OXIDASE5b*, **(C)** *HvBG8*—*β-GLUSIDASE8*, **(D)** *HvBG4*, **(E)** *HvSnRK2.1*—*SNF1-RELATED PROTEIN KINASE 2.1*, **(F)** *HvPYL5*—*PYRABACTIN RESISTANCE 1-LIKE 5*, and **(G)** *HvPP2C4*—*PROTEIN PHOSPATASE 2C 4*. The statistical significance was estimated by T-test between analyzed genotypes—*P ≤ 0.05, **P ≤ 0.01, ***P ≤ 0.001 and between analyzed points—^#^P ≤ 0.05, ^##^P ≤ 0.01, ^###^P ≤ 0.001. **(H)** Endogenous ABA content on 25 DAS under drought in *hvabi5.d* and “Sebastian”. The statistical significance was estimated by T-test between analyzed genotypes—*P ≤ 0.05, **P ≤ 0.01, ***P ≤ 0.001.

The balance between ABA biosynthesis and ABA catabolism is an important factor in activation of stress response (Chan, 2012). *9-cis-epoxycarotenoid dioxygenase-like* (*HvNCED1*) and *ABSCISIC ALDEHYDE OXIDASE 5b* (*HvAO5b*) encode key enzymes in ABA biosynthesis. Under optimal water supply (10 DAS) there were no significant differences between the expression level of *HvNCED1* and *HvAO5b* in WT and mutant (**Figures 10A, B**). Drought stress induced expression of *HvNCED1* in both genotypes, however the differences between *hvabi5.d* mutant and WT were prominent. Drought induced 160-fold increase of expression of *HvNCED1* in the mutant and 80-fold-increase in the WT. On the other hand, the expression of *HvAO5b* gene encoding the last step of ABA biosynthesis raised to the higher level in the WT than in mutant, 55-fold and 20-fold compared to control conditions, respectively.

ABA can be also pooled in ABA-glucose esters (Xu et al., 2014). We analyzed the expression of barley *β-GLUCOSIDASEs* genes, *HvBG8* and *HvBG4*, responsible respectively, for de-conjugation and conjugation of ABA-glucose esters (**Figures 10C, D**). Under optimal water conditions expression of *HvBG8* was very low and did not differ between *hvabi5.d* and WT. In response to drought stress, a 500-fold increase of *HvBG8* expression was observed in the WT and remarkably, a 2,000-fold increase in the mutant. The expression of *HvBG4* showed a different pattern of expression than *HvBG8*, as drought treatment drastically decreased its transcription level in both genotypes. Under optimal water conditions the *hvabi5.d* mutant showed a 5.5-fold higher expression of *HvBG4* compared to “Sebastian”, while after drought treatment *HvBG4* was down-regulated to a greater extent in the mutant (−3,300-fold) than in the WT (−91-fold).

The expression analysis of ABA metabolism genes demonstrated that their regulation differed in *hvabi5.d* and its WT parent. It raised the possibility that HvABI5 might be engaged in the fine tuning of ABA signaling by a feedback regulation between biosynthetic and signaling events. To test this hypothesis the expression of core ABA signaling genes: *SNF1-RELATED PROTEIN KINASE2.1* (*HvSnRK2.1*), *PYRABACTIN RESISTANCE 1-LIKE 5* (*HvPYL5*), and *PROTEIN PHOSPATASE2C4* (*HvPP2C4*) was evaluated in the *hvabi5.d* and WT before and after drought treatment (**Figures 10E–G**). *HvSnRK2.1* and *HvPYL5* were up-regulated in the *hvabi5.d* compared to the WT already under optimal water conditions. After drought stress the down-regulation of *HvPYL5* was observed and no differences between both genotypes were noticed. Interestingly, in response to drought the level of *HvSnRK2.1* and *HvPP2C4* expression was significantly higher in *hvabi5.d* than in the WT and the exceptional 3000-fold change of *HvSnRK2.1* expression in the mutant, 20 times higher than in “Sebastian” was noted. We discuss these results in the further section of the paper.

### Endogenous ABA Content Is Higher in *hvabi5.d* Than in WT Under Drought

Expression of the key ABA metabolism and signaling genes was induced to much higher level in *hvabi5.d* than in WT under drought. Therefore, we presumed that endogenous ABA level might differ in the mutant compared to its parent cultivar. To answer this question, we measured ABA content in plants of both genotypes grown under optimal water conditions and exposed to 10-d drought stress. ABA concentration was below detection level under optimal water supply in “Sebastian” and *hvabi5.d*, whereas in response to drought ABA accumulation increased in both genotypes. Interestingly, the drought stress caused the two-fold higher increase of endogenous ABA content in the *hvabi5.d* than in the WT (**Figure 10H**).

### Promoters of ABA Biosynthesis and Signaling Genes Contain AtABI5 Binding Motifs

ABI transcription factors (AtABI4 and AtABI5) were shown to be involved in a feedback regulation of ABA biosynthesis and signaling genes through direct binding to their promoters and activating their expression (Shu et al., 2016; Wang et al., 2019). Taking into account the changed expression pattern of ABA biosynthesis and signaling genes in *hvabi5.d* under drought, we analyzed transcription factor binding sites in their promoters. We found that promoters of *HvNCED1*, *HvSnRK2.1*, and *HvPP2C4* are enriched in motifs recognized by AtABI5 (**Supplementary Material S12**). Therefore, *HvABI5* might be involved in a feedback regulation of the ABA pathway to ensure the proper amplification of ABA signal in the presence of drought.

### *hvabi5.d* Showed Faster Stomata Closure and Changed Expression of ABA-Related Genes Under ABA Treatment

**Figure 11:**
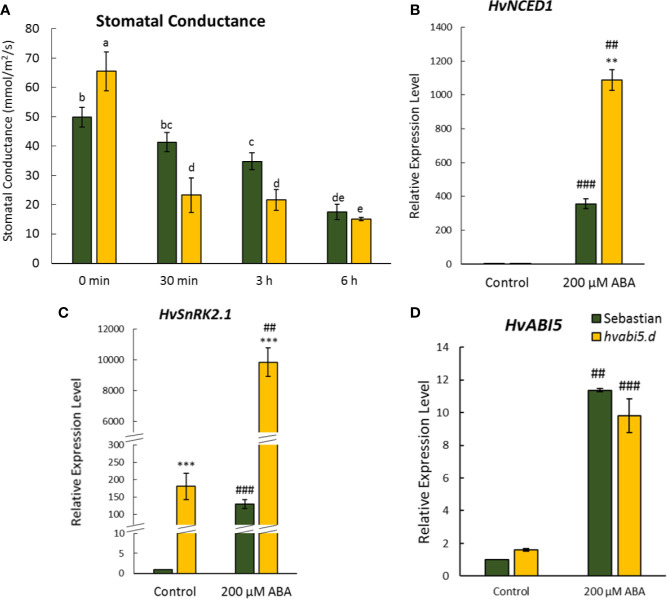
Response of *hvabi5.d* to ABA spraying. **(A)** Stomatal conductance of *hvabi5.d* and its WT parent “Sebastian” after ABA treatment. The statistical analysis was performed using the two-way ANOVA (P ≤ 0.05) followed by Tukey’s honestly significant difference test (Tukey HSD-test) (P ≤ 0.05) to assess the differences between different growth conditions and between genotypes. Statistically significant differences (P ≤ 0.05) are marked by different letters. The expression of **(B)** *HvNCED1*—*CAROTENOID CLEAVAGE DIOXYGENASE1* **(C)** *HvSnRK2.1*—*SNF1-RELATED PROTEIN KINASE 2.1*, and **(D)** *HvABI5*—*ABA INSENSITIVE5* in *hvabi5.d* and its WT 3 h after ABA treatment. The statistical significance was estimated by T-test between analyzed genotypes—**P ≤ 0.01, ***P ≤ 0.001 and between analyzed points—^##^P ≤ 0.01, ^###^P ≤ 0.001.

To confirm if *HvABI5* regulates barley response to drought in the ABA-dependent way, we analyzed stomatal conductance and expression of the selected ABA-related genes in response to exogenously applied ABA. In order to test whether *hvabi5.d* stomata are sensitive to exogenously applied ABA, we measured stomatal conductance 30 min, 3, and 6 h after ABA spraying (**Figure 11A**). The reaction of *hvabi5.d* stomata to ABA was noticed faster than of the WT, already 30 min after treatment. Both genotypes showed the reduction of stomatal conductance 3 and 6 h after ABA treatment, however, 3 h after treatment the stomatal conductance was almost twofold lower in *hvabi5.d* than in WT. These results indicate that mutant stomata were more sensitive to ABA than stomata of its WT parent. Thus, to check the ABA-dependent response of *hvabi5.d* and the WT, we analyzed the expression of ABA biosynthesis gene, *HvNCED1*, and ABA signaling gene, *HvSnRK2.1*, in leaves of both genotypes 3 h after ABA treatment. It should be underlined that drought treatment activated transcription of both genes to the much higher levels in the *hvabi5.d* mutant than in “Sebastian”. As previously described, the expression of *HvSnRK2.1* was higher in *hvabi5.d* than in the WT under control conditions. The ABA treatment highly induced *HvNCED1* and *HvSnRK2.1* expression in both genotypes, nevertheless the expression of *HvNCED1* and *HvSnRK2.1* was much higher in *hvabi5.d* compared to WT, 3 and 76 times, respectively (**Figures 11B, C**). ABA also activated *HvABI5* expression, however we observed no differences between *hvabi5.d* and “Sebastian” (**Figure 11D**).

## Discussion

Here, we report the first barley mutant (*hvabi5.d*) carrying a missense mutation in *HvABI5* gene (Ensembl Plants, Gene ID HORVU5Hr1G068230). HvABI5 belongs to the ABA-dependent bZIP transcription factors that in response to abiotic stresses regulate directly expression of genes containing ABRE motifs in their promoters (Kagaya et al., 2002; Kobayashi et al., 2008; Hossain et al., 2010a; Yan et al., 2012; Piao et al., 2019). Of the Arabidopsis bZIP genes with ABRE motifs, HvABI5 appears to be more related to AtABFs/AtAREBs than to AtABI5. Both, AtABI5 and AtABFs/AtAREBs regulate plant response to abiotic stresses including drought, although at different developmental stages. AtABI5 takes part in plant response to stress mostly at the germination and seedling stages (Finkelstein and Lynch, 2000; Carles et al., 2002; Bi et al., 2017), while AtABFs/AtAREBs act mostly during vegetative growth and their role seems to be highly redundant (Fujita et al., 2005; Yoshida et al., 2010; Yoshida et al., 2015). In our phylogenetic analysis HvABI5 grouped more closely to AtABFs/AtAREBs than to AtABI5, nevertheless the level of the sequence identity between HvABI5 and AtABFs/AtAREBs and between HvABI5 and AtABI5 were very similar. Although we cannot indicate the functional ortholog of HvABI5 in Arabidopsis, it is possible that barley HvABI5 might share common functions with both, AtABFs/AtAREBs and AtABI5. This presumption is supported by the fact that HvABI5 shows the highest level of similarity to monocot bZIPs such as wheat wABI5, rice TRAB1, OsABF4 and OsABF2, and maize ZmABI5, which act as ABA-dependent transcription factors. wABI5, TRAB1, and OsABF2 were described as positive regulators of response to abiotic stresses, including drought in vegetative tissues of these species (Kagaya et al., 2002; Kobayashi et al., 2008; Hossain et al., 2010a). Close phylogenetic relationship of HvABI5 with wABI5, TRAB1, and OsABF2 enhanced our hypothesis that HvABI5 may act as the ABA-dependent regulator of drought response in barley vegetative tissues.

According to the *in silico* analysis and alignment with AtABI5, R274K substitution caused by G1751A mutation in the *hvabi5.d* is located close to the ubiquitination site in the ABI5 protein. The analyzed mutation can generate or diminish the site of post-translational modification in HvABI5, which in turn may change its activity. In this study we confirmed that barley *ABI5* regulates ABA response at early developmental stages, as *hvabi5.d* mutant showed a significantly reduced ABA sensitivity during seed germination and root growth. Similar reaction was reported for Arabidopsis mutants in *AtABI5* and *AtABFs*/*AtAREBs* genes (Finkelstein, 1994; Carles et al., 2002; Yoshida et al., 2010) and rice mutant in *OsABF2* gene (Hossain et al., 2010a). During seedling development in hydroponic conditions in the presence of ABA, *hvabi5.d* was able to accumulate more proline than its parent cv. “Sebastian” and showed the reduced efficiency of photosynthesis. Moreover, we observed a faster stomatal closure in the mutant compared to WT after ABA spraying of 10-d old seedlings. The results obtained after ABA treatment gave us premises to presume that *HvABI5* is involved in the regulation of ABA signaling in barley.

### *hvabi5.d* Showed a Water-Saving Mechanism in Response to Severe Drought Stress

*hvabi5.d* showed a drought-tolerant phenotype manifested mainly by the ability to store water more efficiently than its WT parent “Sebastian”. Our experiment revealed that the higher RWC in *hvabi5.d* leaves after severe drought stress might result from a better membrane protection and a faster stomatal closure. The cell membrane stability expressed as percentage of EL is an indicator of the level of tolerance to water stress in plants (Bajji et al., 2002). Our results showed that the cellular membranes of the *hvabi5.d* were less damaged than the WT after drought treatment.

Better membrane performance in *hvabi5.d* under drought stress might result from the more efficient activation of protection mechanisms in the mutant, as revealed by transcriptomic analysis. In response to drought, the *hvabi5.d* showed increased expression of genes encoding late embryogenesis abundant proteins (LEAs) and HSPs (heat shock 70 kDa protein 3, Heat shock 70 kDa protein 15). The LEA and HSP proteins enable adaptation to abiotic stress through protection of proteins and membrane structure (Vinocur and Altman, 2005; Shinozaki and Yamaguchi-Shinozaki, 2007; Bhatnagar-Mathur et al., 2008). AtABI5, AtABFs/AtAREBs, and their monocot homologs were shown to regulate expression of *LEA* genes (Kobayashi et al., 2008; Yoshida et al., 2010; Yan et al., 2012; Su et al., 2016). In barley, HvABI5 was shown to directly activate *HVA1* and *HVA22* genes that encode LEA proteins that ensure tolerance to low water availability (Hong et al., 1992; Shen et al., 2001). It was also shown that *HVA1* is regulated by other transcription factor, HvDRF1 (Xue and Loveridge, 2004). We observed the increased expression of *HVA1*, *HVA22*, and *HvDRF1* in *hvabi5.d* under drought. Furthermore, the global transcriptome analysis also revealed a higher expression of *HVA22 homolog A* and *ethylene-responsive transcription factor 4/dehydration responsive element binding protein 3* (*HvDRF1*). Therefore, the increased activation of *HVA1* and *HVA22* in the mutant may result simultaneously from the changed activity of HvABI5 protein and the higher expression of *HvDRF1*. Taken together, the higher water content and better membrane stability of *hvabi5.d* under water stress, compared to the WT, can be associated with up-regulation of *LEA* and *HSP* genes.

In addition, better membrane stability of *hvabi5.d* and its drought tolerant phenotype might be related to a higher content of flavonoids: flavonols and anthocyanins. Flavonoids protect plants against ROS under abiotic stress (Di Ferdinando et al., 2012; Davies et al., 2018). The higher expression of genes encoding flavonoid biosynthesis enzymes (anthocyanidin reductase, flavanone 3-hydroxylase, chalcone-flavonone isomerase, and MYB domain protein 30) observed in our study could explain the higher flavonoid content in *hvabi5.d* under drought. The relationship between *AtABI5* and anthocyanins biosynthesis was previously observed in Arabidopsis by Brocard et al. (2002). Another mechanism contributing to increased drought tolerance of *hvabi5.d* could be related to detoxification enzymes, that similarly to flavonoids protect against ROS under abiotic stress (Anjum et al., 2016; Mishra and Sharma, 2019). The increased expression of genes encoding PRXs (peroxidase superfamily proteins) and SOD ([Cu-Zn] 2) could alleviate drought-induced damages in *hvabi5.d*. It was shown that ABI5 directly regulated expression of genes encoding detoxification enzymes in Arabidopsis and barley (Bi et al., 2017; Ishibashi et al., 2017). Moreover, over-expression of *ZmABI5*, a maize homolog of *HvABI5*, activated expression of *ASCORBATE PEROXIDASE (APX)* under drought (Yan et al., 2012).

Another physiological trait that ensures a low water loss is stomatal closure in response to water scarcity. We observed a lower stomatal conductance in *hvabi5.d* mutant than in WT at the onset of drought stress (13 and 15 DAS), but not after application of severe drought (25 DAS). The faster stomata closure can also contribute to the increased drought tolerance of *havbi5.d*. We discuss the possible mechanism underlying this process in the further part of *Discussion*.

The altered HvABI5 function in *hvabi5.d* mutant negatively influenced the photosynthesis efficiency and chlorophyll content under drought stress. We presumed that impaired photosynthesis of *hvabi5.d* after drought treatment could be the effect of both, the lower stomatal conductance at the onset of drought stress and the lower chlorophyll content. The role of ABA and ABA signaling components in chlorophyll degradation has been well described (Yang et al., 2014; Gao et al., 2016). In Arabidopsis, the involvement of AtABI5 in regulation of photosynthesis was shown in terms of repression of *ABA-RESPONSE PROTEIN* (*ABR*) responsible for protection of photosynthesis proteins (Su et al., 2016). Furthermore, AtABI5 directly promoted expression of chlorophyll catabolism-related genes (*STAYGREEN 1*—*SGR1* and *NON-YELLOW COLORING 1*—*NYC1*) (Sakuraba et al., 2014). Similarly, AtABFs/AtAREBs were shown to regulate directly genes associated with chlorophyll degradation: *SGR1*, *NYC1* and *PHEOPHORBIDE A OXYGENASE* (PAO) (Gao et al., 2016). In rice OsABF4 activated directly expression of *OsSGR1* and *OsNYC1* (Piao et al., 2019). Our transcriptomic data revealed the lower expression level of both, photosynthesis-related genes (*chaperonin-like RbcX proteins*, *TPR-containing protein*) and genes associated with chlorophyll function (*chlorophyll A/B binding protein 3*, *high chlorophyll fluorescence phenotype 173*) in *hvabi5.d* under drought.

It should be noted that *hvabi5.d* is the first *ABI5* mutant evaluated in detail in terms of tolerance to drought stress. Arabidopsis *Atabi5-1* mutant has not been characterized in this respect, it was only described as “not-wilty” in databases (Finkelstein and Lynch, 2000; http://www.arabidopsis.org, AT2G36270). The same lack of data applies to *abi5* mutants of *Medicago truncatula* and *Pisum sativum* (Zinsmeister et al., 2016). The only mutants studied in this respect: *Atabf4*/*Atareb2*, *Atabf3*, *Osabf1*, and *Osabf2* showed a lower survival rate after drought application (Hossain et al., 2010a; Hossain et al., 2010b; Yoshida et al., 2010). The changed expression of *AtABI5* under drought stress (Brocard et al., 2002), a drought tolerant phenotype of plants over-expressing *wABI5* and *AtABFs*/*AtAREBs* genes (Kang et al., 2002; Fujita et al., 2005; Kobayashi et al., 2008) and drought sensitive phenotype of lines over-expressing *ZmABI5* (Yan et al., 2012) are in agreement with the role of barley ABI5 in drought response presented in this study.

### Drought Response of *hvabi5.d* Is the ABA-Dependent Process

Regarding *hvabi5.d* response to ABA at early seedling stage and its drought tolerant phenotype we presumed that *HvABI5* regulated drought response in the ABA-dependent way. To verify this hypothesis, we analyzed expression of the ABA pathway genes under drought and ABA. Among two genes encoding key enzymes in ABA biosynthesis (*HvNCED1* and *HvAo5b*) analyzed in our study, only *HvNCED1*, which is a rate-limiting enzyme for ABA biosynthesis, showed a much higher expression in *hvabi5.d*, compared to WT, in response to drought and ABA. The expression of *HvAo5b* increased after drought stress in both genotypes. However, the much lower transcription level of *HvAo5b* in *hvabi5.d* than in the WT might level out the effect of the high *HvNCED1* expression in the mutant. Furthermore, *hvabi5.d* showed, respectively, higher and lower expression of *HvBG8* and *HvBG4* after drought. *HvBG8* is responsible for ABA de-conjugation, whereas *HvBG4* takes part in ABA conjugation. It suggests that ABA is more effectively released from ABA glucosyl esters in *hvabi5.d* in response to drought. This mechanism might be important for the higher drought tolerance of the mutant.

We investigated also the expression of genes encoding the core ABA signaling, such as *HvPYL5*, *HvPPC4*, and *HvSnRK2.1*. According to the basic model of ABA signal pathway, in the presence of endogenous ABA PYR/PYLs receptors interact with PP2Cs and inhibit phosphatase activity, allowing SnRK2 activation and phosphorylation of target proteins, among them ABI5. In our study, the down-regulation of *HvPYL5* and up-regulation of *HvPP2C4* was observed in both, *hvabi5.d* and WT, in response to drought. However, the increase of *HvPP2C4* expression was twofold higher in the mutant than in the WT. Moreover, we noticed substantially higher activation of *HvSnRK2.1* in *hvabi5.d* than in WT under drought and ABA. Increased induction of *HvSnRK2.1* expression was also observed by Seiler et al. (2014) in drought-tolerant line of barley. It can be presumed that the increased level of *HvSnRK2.1* expression in *hvabi5.d* enables the amplification of ABA signaling and activation of ABA-related genes.

Drought-induced ABA biosynthesis is one of the main processes of plant adaptation to stress (Yoshida et al., 2019). The increased expression of ABA metabolism genes in *havbi5.d* resulted in the higher ABA content in the mutant compared to the WT. It indicates that *HvABI5* takes part in triggering ABA accumulation and subsequent activation of ABA signaling, which in turn ensures response to drought through many mechanisms, among them stomatal closure.

Together, these findings demonstrated the role of *HvABI5* in the feedback regulation of components of ABA biosynthesis and signaling under drought stress. We showed a possibility of HvABI5 engagement in a feedback up-regulation of ABA synthesis (via *HvNCED1*) that may help to amplify the ABA signal under stress and simultaneous up-regulation of PP2C4 that might contribute to avoiding the over-amplification of the stress signal in order to maintain adaptation and performance under stress. Recently AtABF1, AtABF2, AtABF3, AtABF4, and AtABI5 were shown to bind directly to promoters of *ABI1* and *ABI2*, which encode PP2C type phosphatases. This mechanism ensures negative feedback loop in ABA signaling (Wang et al., 2019). Similarly, the up-regulation of *HvSnRK2.1* in the *hvabi5.d* mutant raises the possibility of *HvABI5* engagement in the feedback regulation of this kinase activity. It should be underlined that we found ABI5 binding elements in the promoters of *HvNCED1*, *HvPP2C4*, and *HvSnRK2.1*. Thus, it might be possible that HvABI5 directly regulates expression of these genes. Simultaneous upregulation of ABA synthesis *via* induction of *HvNCED1*, and ABA signaling *via* induction of *HvPP2C4* and *HvSnRK2.1* in *hvabi5.d* mutant in response to drought stress indicate a fine-tuned control of the amplification of stress signaling and needs further investigations.

Our results strongly support the hypothesis that drought response of *hvabi5.d* is the ABA-dependent process. Yet, *hvabi5.d* showed reduced ABA sensitivity at germination stage. It indicates that ABA-dependent function of *HvABI5* at seed germination differs from its role in the regulatory pathway involving *HvABI5* under drought stress. *HvABI5* expression is not restricted to a short developmental window during seed germination and early seedling growth, as shown in Arabidopsis (Maia et al., 2014). It is possible that HvABI5 has acquired new functions during evolution in barley. It has been proved that genes responsible for plant fitness evolve faster than core metabolism genes (Nelissen et al., 2014). Furthermore, many abiotic stress tolerance mechanisms of monocots can differ from those in Arabidopsis (Tester and Bacic, 2005). In Arabidopsis, ABA-mediated response to abiotic stress is regulated by *AtABI5* in seeds and *AtABFs/AtAREBs* in leaves and roots at vegetative stage. However, function of *AtABFs/AtAREBs* in ABA signaling is highly redundant. Here, we indicate that due to evolution events *HvABI5* shares some function with *AtABI5* and *AtABFs/AtAREBs* and acts simultaneously as the ABA-dependent regulator at the seed germination stage and in response to drought in seedling vegetative tissues. Contrary to *AtABFs/AtAREBs*, the role of *HvABI5* under drought seems to be individual since *hvabi5.d* shows drought tolerant phenotype, while triple *Atabf2*/*areb1 Atabf4*/*areb2 Atabf3* mutant exhibited significantly disturbed phenotype under drought (Yoshida et al., 2010). On the other side, similarly to *AtABFs/AtAREBs*, *HvABI5* acts in the ABA signaling as a regulator of *LEAs* expression, chlorophyll catabolism and stomatal closure, and takes part in the feedback regulation of ABA pathway. In monocots, *wABI5*, *OsABF2*, *TRAB1*, and *ZmABI5* regulate ABA-mediated drought response by activation of stress responsive genes in seedling vegetative tissues (Kagaya et al., 2002; Kobayashi et al., 2008; Hossain et al., 2010a; Yan et al., 2012). Furthermore, *OsABF2* and *ZmABI5* were also shown to be active in seeds (Hossain et al., 2010a; Zhang et al., 2019). We revealed that function of HvABI5 as the ABA-dependent regulator of stress response is similar to its close monocot homologs.

Finally, we want to address the issue of the impact of possible background mutations on *hvabi5.d* phenotype. Taking into account that *hvabi5.d* was identified in the TILLING population derived from chemical mutagenesis, such a possibility cannot be ruled out. The best way to prove that the observed phenotypic change results from the identified mutation is to complement the mutated allele through genetic transformation with the WT gene. However, in barley the effective transformation is restricted merely to a single cultivar, “Golden Promise”. Cultivar “Sebastian”—the parent of *hvabi5.d* mutant turn out to be recalcitrant to *Agrobacterium* transformation in our preliminary experiments (data not shown). However, it should be underlined that *hvabi5.d* was identified as one of four mutants with different *hvabi5* alleles that performed better than WT after drought treatment in our preliminary screening. We examined seven independent TILLING mutants carrying different alleles in the *HvABI5* gene for their response to drought stress. Four of these mutants, including *hvabi5.d*, showed increased water content after drought treatment in the RWC assay. Three other tested alleles showed no significant differences in RWC value compared to WT, which indicates that these missense mutations did not evoke any significant changes in function of HvABI5 protein.

Furthermore, we developed *hvabi5.d* lines after two back-crosses to the parent cultivar “Sebastian” to clean the mutant from the possible background mutations. The created F_4_BC_2_ lines with *hvabi5.d* allele were then analyzed for the basic physiological traits related to drought response (RWC, flavonol and anthocyanin content, stomatal conductance) after exposure to drought stress. We also examined expression of HvABI5-related genes (*HVA1, HVA22, HvDRF1*) and genes involved in ABA biosynthesis, metabolism, and signaling (*HvNCED1, HvBG8, HvSnRK2*, and *HvPP2C4*) in these lines. All analyzed physiological parameters, as well as expression of all analyzed genes in the *hvabi5.d* F_4_BC_2_ were similar to those observed in the mutant exposed to drought, described in this study (**Supplementary Material S13**). Taken together our analysis clearly confirmed the association between *HvABI5* function and regulation of drought stress response in barley. It should be noted that another approach to linking phenotype and a causative mutation i.e., analysis of their co-segregation in a segregating F_2_ population cannot be performed for qualitative traits such as drought tolerance. In the case of *hvabi5.d* it was not possible to distinguish between mutant and WT phenotype in zero-one categories. All tests used in the presented study required biological replications, therefore the analysis of segregation in F_2_ generation could not be performed on the basis of single plants phenotyping.

## Conclusions

The presented results bring the set of new data regarding the function of *HvABI5* in the ABA signaling during drought response in barley. We found that *HvABI5* takes part in regulation of processes associated with drought stress tolerance, such as membrane protection, flavonoid accumulation, and stomatal closure. *HvABI5* activates expression of stress-responsive genes, which ensure plant adaptation to drought stress. We proved that *HvABI5* regulates drought response in the ABA-dependent way. Our data indicate that *HvABI5* can directly activate ABA biosynthesis and signaling genes and therefore it ensures the proper amplification of ABA signal under drought. We also found that the ABA-dependent regulatory role of *HvABI5* during drought response differs from its role at seed germination. Together, these findings increase our understanding of ABI5-dependent modulation of plant response to the abiotic stress. Further analysis is needed to confirm the interaction between HvABI5 and promoters of its putative target genes.

## Data Availability Statement

The datasets presented in this study can be found in online repositories. The names of the repository/repositories and accession number(s) can be found below:

http://www.ebi.ac.uk/arrayexpress/experiments/E-MTAB-9072.

## Author Contributions

IS conceived and supervised the project and contributed to the development of the TILLING platform, IS and AD-G designed the study and contributed to the analysis of results, AC and AD-G performed drought experiment, AC conducted physiological assays and gene expression analysis, and AD-G analyzed photosynthesis and microarray data. MK performed TILLING screen. AC, AD-G, and IS wrote and edited the manuscript.

## Funding

This work was supported by the European Regional Development Fund through the Innovative Economy for Poland 2007-2013, project WND-POIG.01.03.01-00-101/08 POLAPGEN-BD “Biotechnological tools for breeding cereals with increased resistance to drought”, task 22 and by the National Science Centre, Poland, project PRELUDIUM 2017/25/N/NZ9/01941 “The role of HvABI5 transcription factor in spring barley (*Hordeum vulgare* L.) response to drought stress”.

## Conflict of Interest

The authors declare that the research was conducted in the absence of any commercial or financial relationships that could be construed as a potential conflict of interest.

## References

[B1] Agarwal P. K. Shukla P. S. Gupta K. Jha B. (2013). Bioengineering for salinity tolerance in plants: state of the art. Mol. Biotechnol. 54, 102–123. 10.1007/s12033-012-9538-3 22539206

[B2] Albertos P. Romero-Puertas M. C. Tatematsu K. Mateos I. Sánchez-Vicente I. Nambara E. (2015). S-nitrosylation triggers ABI5 degradation to promote seed germination and seedling growth. Nat. Commun. 6, 8669. 10.1038/ncomms9669 26493030 PMC4639896

[B3] Al-Momany B. Abu-Romman S. (2014). Cloning and molecular characterization of a flavin-dependent oxidoreductase gene from barley. J. Appl. Genet. 55, 457–468. 10.1007/s13353-014-0227-8 24961571

[B4] Anjum N. A. Sharma P. Gill S. S. Hasanuzzaman M. Khan E. A. Kachhap K. (2016). Catalase and ascorbate peroxidase—representative H2O2-detoxifying heme enzymes in plants. Environ. Sci. Pollut. R. 23, 19002–19029. 10.1007/s11356-016-7309-6 27549233

[B5] Bailey-Serres J. Parker J. E. Ainsworth E. A. Oldroyd G. E. Schroeder J. II (2019). Genetic strategies for improving crop yields. Nature 575, 109–118. 10.1038/s41586-019-1679-0 31695205 PMC7024682

[B6] Bajji M. Kinet J. M. Lutts S. (2002). The use of the electrolyte leakage method for assessing cell membrane stability as a water stress tolerance test in durum wheat. Plant Growth Regul. 36, 61–70. 10.1023/A:1014732714549

[B7] Bandurska H. Gniazdowska-Skoczek H. (1995). Cell membrane stability in two barley genotypes under water stress conditions. Acta Soc Bot. Pol. 64, 29–32. 10.5586/asbp.1995.005

[B8] Bensmihen S. To A. Lambert G. Kroj T. Giraudat J. Parcy F. (2004). Analysis of an activated ABI5 allele using a new selection method for transgenic Arabidopsis seeds. FEBS Lett. 561, 127–131. 10.1016/S0014-5793(04)00148-6 15013763

[B9] Bhatnagar-Mathur P. Vadez V. Sharma K. K. (2008). Transgenic approaches for abiotic stress tolerance in plants: retrospect and prospects. Plant Cell Rep. 27, 411–424. 10.1007/s00299-007-0474-9 18026957

[B10] Bi C. Ma Y. Wu Z. Yu Y. T. Liang S. Lu K. (2017). Arabidopsis ABI5 plays a role in regulating ROS homeostasis by activating CATALASE 1 transcription in seed germination. Plant Mol. Biol. 94, 197–213. 10.1007/s11103-017-0603-y 28391398 PMC5437177

[B11] Brocard I. M. Lynch T. J. Finkelstein R. R. (2002). Regulation and role of the Arabidopsis *ABSCISIC ACID-INSENSITIVE 5* gene in abscisic acid, sugar, and stress response. Plant Physiol. 129, 1533–1543. 10.1104/pp.005793 12177466 PMC166741

[B12] Cai S. Chen G. Wang Y. Huang Y. Marchant D. B. Wang Y. (2017). Evolutionary conservation of ABA signaling for stomatal closure. Plant Physiol. 174, 732–747. 10.1104/pp.16.01848 28232585 PMC5462018

[B13] Carillo P. Y. Gibon and PrometheusWiki contributors (2017). Protocol: extraction and determination of proline. PrometheusWiki 2011, 1–4.

[B14] Carles C. Bies-Etheve N. Aspart L. Léon-Kloosterziel K. M. Koornneef M. Echeverria M. (2002). Regulation of *Arabidopsis thaliana Em* genes: role of ABI5. Plant J. 30, 373–383. 10.1046/j.1365-313X.2002.01295.x 12000684

[B15] Casaretto J. Ho T. H. D. (2003). The transcription factors HvABI5 and HvVP1 are required for the abscisic acid induction of gene expression in barley aleurone cells. Plant Cell 15, 271–284. 10.1105/tpc.007096 12509536 PMC143496

[B16] Casaretto J. A. Ho T. H. D. (2005). Transcriptional regulation by abscisic acid in barley (Hordeum vulgare L.) seeds involves autoregulation of the transcription factor HvABI5. Plant Mol. Biol. 57, 21–34. 10.1007/s11103-004-6520-x 15821866

[B17] Chan Z. (2012). Expression profiling of ABA pathway transcripts indicates crosstalk between abiotic and biotic stress responses in Arabidopsis. Genomics 100, 110–115. 10.1016/j.ygeno.2012.06.004 22709556

[B18] Chomczyński P. Sacchi N. (1987). Single-step method of RNA isolation by acid guanidinium thiocyanate-phenol-chloroform extraction. Anal. Biochem. 162, 156–159. 10.1016/0003-2697(87)90021-2 2440339

[B19] Daszkowska-Golec A. Skubacz A. Marzec M. Slota M. Kurowska M. Gajecka M. (2017). Mutation in HvCBP20 (Cap Binding Protein 20) Adapts Barley to Drought Stress at Phenotypic and Transcriptomic Levels. Front. Plant Sci. 8:942. 10.3389/fpls.2017.00942 28626467 PMC5454077

[B20] Daszkowska-Golec A. (2016). “The role of abscisic acid in drought stress: how aba helps plants to cope with drought stress,” in Drought Stress Tolerance in Plants, vol. 2 . Eds. Hossain M. A. Wani S. H. Bhattachajee S. Burritt D. J. Tran L. S. P. (Cham: Springer International Publishing), 123–151.

[B21] Davies K. M. Albert N. W. Zhou Y. Schwinn K. E. (2018). Functions of flavonoid and betalain pigments in abiotic stress tolerance in plants. Annu. Plant Rev. Online, 1–41 (1), 21–62. 10.1002/9781119312994.apr0604

[B22] Dawson I. K. Russell J. Powell W. Steffenson B. Thomas W. T. Waugh R. (2015). Barley: a translational model for adaptation to climate change. New Phytol. 206, 913–931. 10.1111/nph.13266 25605349

[B23] Dejonghe W. Okamoto M. Cutler S. R. (2018). Small molecule probes of ABA biosynthesis and signaling. Plant Cell Physiol. 59, 1490–1499. 10.1093/pcp/pcy126 29986078

[B24] Di Ferdinando M. Brunetti C. Fini A. Tattini M. (2012). “Flavonoids as antioxidants in plants under abiotic stresses,” in Abiotic Stress Responses in Plants. Eds. Ahmad P. Prasad M. N. V. (New York: Springer New York), 159–179.

[B25] FAO (2018). FAOSTAT Database Collections. Food and Agriculture Organization of the United Nations. Available at: http://faostat3.fao.org/ (Accessed March 19, 2020).

[B26] Finkelstein R. R. Lynch T. J. (2000). The Arabidopsis abscisic acid response gene ABI5 encodes a basic leucine zipper transcription factor. Plant Cell 12, 599–609. 10.1105/tpc.12.4.599 10760247 PMC139856

[B27] Finkelstein R. Gampala S. S. Lynch T. J. Thomas T. L. Rock C. D. (2005). Redundant and distinct functions of the ABA response loci ABA-INSENSITIVE (ABI) 5 and ABRE-BINDING FACTOR (ABF) 3. Plant Mol. Biol. 59, 253–267. 10.1007/s11103-005-8767-2 16247556

[B28] Finkelstein R. R. (1994). Mutations at two new Arabidopsis ABA response loci are similar to the *abi3* mutations. Plant J. 5, 765–771. 10.1046/j.1365-313X.1994.5060765.x

[B29] Fujita Y. Fujita M. Satoh R. Maruyama K. Parvez M. M. Seki M. (2005). AREB1 is a transcription activator of novel ABRE-dependent ABA signaling that enhances drought stress tolerance in Arabidopsis. Plant Cell 17, 3470–3488. 10.1105/tpc.105.035659 16284313 PMC1315382

[B30] Furihata T. Maruyama K. Fujita Y. Umezawa T. Yoshida R. Shinozaki K. (2006). Abscisic acid-dependent multisite phosphorylation regulates the activity of a transcription activator AREB1. Proc. Natl. Acad. Sci. U. S. A. 103, 1988–1993. 10.1073/pnas.0505667103 16446457 PMC1413621

[B31] Gao S. Gao J. Zhu X. Song Y. Li Z. Ren G. (2016). ABF2, ABF3, and ABF4 promote ABA-mediated chlorophyll degradation and leaf senescence by transcriptional activation of chlorophyll catabolic genes and senescence-associated genes in Arabidopsis. Mol. Plant 9, 1272–1285. 10.1016/j.molp.2016.06.006 27373216

[B32] Gilliham M. Able J. A. Roy S. J. (2017). Translating knowledge about abiotic stress tolerance to breeding programmes. Plant J. 90, 898–917. 10.1111/tpj.13456 27987327

[B33] Hong B. Barg R. Ho T. H. D. (1992). Developmental and organ-specific expression of an ABA-and stress-induced protein in barley. Plant Mol. Biol. 18, 663–674. 10.1007/BF00020009 1532749

[B34] Hossain M. A. Cho J. II Han M. Ahn C. H. Jeon J. S. An G. (2010a). The ABRE-binding bZIP transcription factor OsABF2 is a positive regulator of abiotic stress and ABA signaling in rice. J. Plant Physiol. 67, 1512–1520. 10.1016/j.jplph.2010.05.008 20576316

[B35] Hossain M. A. Lee Y. Cho J. II Ahn C. H. Lee S. K. Jeon J. S. (2010b). The bZIP transcription factor OsABF1 is an ABA responsive element binding factor that enhances abiotic stress signaling in rice. Plant Mol. Biol. 72, 557–566. 10.1007/s11103-009-9592-9 20039193

[B36] Huang Y. Sun M. M. Ye Q. Wu X. Q. Wu W. H. Chen Y. F. (2017). Abscisic acid modulates seed germination via ABA INSENSITIVE5-mediated PHOSPHATE1. Plant Physiol. 175, 1661–1668. 10.1104/pp.17.00164 29089393 PMC5717723

[B37] Ishibashi Y. Aoki N. Kasa S. Sakamoto M. Kai K. Tomokiyo R. (2017). The interrelationship between abscisic acid and reactive oxygen species plays a key role in barley seed dormancy and germination. Front. Plant Sci. 8:275. 10.3389/fpls.2017.00275 28377774 PMC5359625

[B38] Jones A. M. (2016). A new look at stress: abscisic acid patterns and dynamics at high–resolution. New Phytol. 210, 38–44. 10.1111/nph.13552 26201893

[B39] Joshi R. Wani S. H. Singh B. Bohra A. Dar Z. A. Lone A. A. (2016). Transcription factors and plants response to drought stress: Current understanding and future directions. Front. Plant Sci. 7:1029. 10.3389/fpls.2016.01029 27471513 PMC4943945

[B40] Kagaya Y. Hobo T. Murata M. Ban A. Hattori T. (2002). Abscisic acid–induced transcription is mediated by phosphorylation of an abscisic acid response element binding factor, TRAB1. Plant Cell 14, 3177–3189. 10.1105/tpc.005272 12468735 PMC151210

[B41] Kalaji H. M. Jajoo A. Oukarroum A. Brestic M. Zivcak M. Samborska I. A. (2016). Chlorophyll *a* fluorescence as a tool to monitor physiological status of plants under abiotic stress conditions. Acta Physiol. Plant 38, 102. 10.1007/s11738-016-2113-y

[B42] Kanai M. Nishimura M. Hayashi M. (2010). A peroxisomal ABC transporter promotes seed germination by inducing pectin degradation under the control of ABI5. Plant J. 62, 936–947. 10.1111/j.1365-313X.2010.04205.x 20345608

[B43] Kang J. Y. Choi H. II Im M. Y. Kim S. Y. (2002). Arabidopsis basic leucine zipper proteins that mediate stress-responsive abscisic acid signaling. Plant Cell 14, 343–357. 10.1105/tpc.010362 11884679 PMC152917

[B44] Kobayashi F. Maeta E. Terashima A. Takumi S. (2008). Positive role of a wheat HvABI5 ortholog in abiotic stress response of seedlings. Physiol. Plant 134, 74–86. 10.1111/j.1399-3054.2008.01107.x 18433415

[B45] Kong Y. Chen S. Yang Y. An C. (2013). ABA-insensitive (ABI) 948 4 and ABI5 synergistically regulate *DGAT1* expression in Arabidopsis seedlings under stress. FEBS Lett. 587, 3076–3082. 10.1016/j.febslet.2013.07.045 23942253

[B46] Liu H. Stone S. L. (2013). Cytoplasmic degradation of the Arabidopsis transcription factor abscisic acid insensitive 5 is mediated by the RING-type E3 ligase KEEP ON GOING. J. Biol. Chem. 288, 20267–20279. 10.1074/jbc.M113.465369 23720747 PMC3711294

[B47] Livak K. J. Schmittgen T. D. (2001). Analysis of relative gene expression data using real-time quantitative PCR and the 2– ΔΔCT method. Methods 25, 402–408. 10.1006/meth.2001.1262 11846609

[B48] Lopez-Molina L. Chua N. H. (2000). A null mutation in a bZIP factor confers ABA-insensitivity in *Arabidopsis thaliana*. Plant Cell Physiol. 41, 541–547. 10.1093/pcp/41.5.541 10929936

[B49] Lopez-Molina L. Mongrand S. Chua N. H. (2001). A postgermination developmental arrest checkpoint is mediated by abscisic acid and requires the ABI5 transcription factor in Arabidopsis. Proc. Natl. Acad. Sci. U. S. A. 98, 4782–4787. 10.1073/pnas.081594298 11287670 PMC31911

[B50] Lynch T. Erickson B. J. Finkelstein R. R. (2012). Direct interactions of ABA-insensitive (ABI)-clade protein phosphatase (PP) 2Cs with calcium-dependent protein kinases and ABA response element-binding bZIPs may contribute to turning off ABA response. Plant Mol. Biol. 80, 647–658. 10.1007/s11103-012-9973-3 23007729

[B51] Maia J. Dekkers B. J. Dolle M. J. Ligterink W. Hilhorst H. W. (2014). Abscisic acid (ABA) sensitivity regulates desiccation tolerance in germinated Arabidopsis seeds. New Phytol. 203, 81–93. 10.1111/nph.12785 24697728

[B52] Martignago D. Rico-Medina A. Blasco-Escaméz D. Fontanet-Manzaneque J. B. Caño-Delgado A. II (2020). Drought resistance by engineering plant tissue-specific responses. Front. Plant Sci. 10:1676. 10.3389/fpls.2019.01676 32038670 PMC6987726

[B53] Mishra P. Sharma P. (2019). “Superoxide Dismutases (SODs) and Their Role in Regulating Abiotic Stress induced Oxidative Stress in Plants,” in Reactive Oxygen, Nitrogen and Sulfur Species in Plants. Eds. Hasanuzzaman M. Fotopoulos V. Nahar K. Fujita M. (Hoboken: John Wiley & Sons Ltd), 53–88.

[B54] Miura K. Lee J. Jin J. B. Yoo C. Y. Miura T. Hasegawa P. M. (2009). Sumoylation of ABI5 by the Arabidopsis SUMO E3 ligase SIZ1 negatively regulates abscisic acid signaling. Proc. Natl. Acad. Sci. U. S. A. 106, 5418–5423. 10.1073/pnas.0811088106 19276109 PMC2664011

[B55] Nakamura S. Lynch T. J. Finkelstein R. R. (2001). Physical interactions between ABA response loci of Arabidopsis. Plant J. 26, 627–635. 10.1046/j.1365-313x.2001.01069.x 11489176

[B56] Nakurte I. Keisa A. Rostoks N. (2012). Development and Validation of a Reversed-Phase Liquid Chromatography Method for the Simultaneous Determination of Indole-3-Acetic Acid, Indole-3-Pyruvic Acid, and Abscisic Acid in Barley (*Hordeum vulgare* L.). J. Anal. Methods Chem. 2012, 103575. 10.1155/2012/103575 22567549 PMC3335325

[B57] Nambara E. Suzuki M. Abrams S. McCarty D. R. Kamiya Y. McCourt P. (2002). A screen for genes that function in abscisic acid signaling in Arabidopsis thaliana. Genetics 161, 1247–1255. 12136027 10.1093/genetics/161.3.1247 PMC1462180

[B58] Nelissen H. Moloney M. Inzé D. (2014). Translational research: from pot to plot. Plant Biotechnol. J. 12, 277–285. 10.1111/pbi.12176 24646295

[B59] Paul S. Roychoudhury A. (2019). Transcript analysis of abscisic acid-inducible genes in response to different abiotic disturbances in two indica rice varieties. Theor. Exp. Plant Phys. 31, 249–272. 10.1007/s40626-018-0131-4

[B60] Piao W. Kim S. H. Lee B. D. An G. Sakuraba Y. Paek N. C. (2019). Rice transcription factor OsMYB102 delays leaf senescence by down-regulating abscisic acid accumulation and signaling. J. Exp. Bot. 70, 2699–2715. 10.1093/jxb/erz095 30825376 PMC6506775

[B61] Qian D. Zhang Z. He J. Zhang P. Ou X. Li T. (2019). Arabidopsis ADF5 promotes stomatal closure by regulating actin cytoskeleton remodeling in response to ABA and drought stress. J. Exp. Bot. 70, 435–446. 10.1093/jxb/ery385 30476276 PMC6322581

[B62] Ramakers C. Ruijter J. M. Deprez R. H. L. Moorman A. F. (2003). Assumption-free analysis of quantitative real-time polymerase chain reaction (PCR) data. Neurosci. Lett. 339, 62–66. 10.1016/S0304-3940(02)01423-4 12618301

[B63] Rapacz M. Stępień A. Skorupa K. (2012). Internal standards for quantitative RT-PCR studies of gene expression under drought treatment in barley (*Hordeum vulgare* L.): the effects of developmental stage and leaf age. Acta Physiol. Plant 34, 1723–1733. 10.1007/s11738-012-0967-1

[B64] Sah S. K. Reddy K. R. Li J. (2016). Abscisic acid and abiotic stress tolerance in crop plants. Front. Plant Sci. 7:571. 10.3389/fpls.2016.00571 27200044 PMC4855980

[B65] Saito S. Uozumi N. (2019). Guard cell membrane anion transport systems and their regulatory components: an elaborate mechanism controlling stress-induced stomatal closure. Plants 8, 9. 10.3390/plants8010009 PMC6359458 30609843

[B66] Sakuraba Y. Jeong J. Kang M. Y. Kim J. Paek N. C. Choi G. (2014). Phytochrome interacting transcription factors PIF4 and PIF5 induce leaf senescence in Arabidopsis. Nat. Commun. 5, 4636. 10.1038/ncomms5636 25119965

[B67] Seiler C. Harshavardhan V. T. Reddy P. S. Hensel G. Kumlehn J. Eschen-Lippold L. (2014). Abscisic acid flux alterations result in differential abscisic acid signaling responses and impact assimilation efficiency in barley under terminal drought stress. Plant Physiol. 164, 1677–1696. 10.1104/pp.113.229062 24610749 PMC3982733

[B68] Shen Q. Chen C. N. Brands A. Pan S. M. Tuan-Hua D. H. (2001). The stress-and abscisic acid-induced barley gene *HVA22*: developmental regulation and homologues in diverse organisms. Plant Mol. Biol. 45, 327–340. 10.1023/A:1006460231978 11292078

[B69] Shinozaki K. Yamaguchi-Shinozaki K. (2007). Gene networks involved in drought stress response and tolerance. J. Exp. Bot. 58, 221–227. 10.1093/jxb/erl164 17075077

[B70] Shu K. Chen Q. Wu Y. Liu R. Zhang H. Wang P. (2016). ABI4 mediates antagonistic effects of abscisic acid and gibberellins at transcript and protein levels. Plant J. 85, 348–361. 10.1111/tpj.13109 26708041

[B71] Signora L. De Smet I. Foyer C. H. Zhang H. (2001). ABA plays a central role in mediating the regulatory effects of nitrate on root branching in Arabidopsis. Plant J. 28, 655–662. 10.1046/j.1365-313x.2001.01185.x 11851911

[B72] Skubacz A. Daszkowska-Golec A. Szarejko I. (2016). The role and regulation of ABI5 (ABA-insensitive 5) in plant development, abiotic stress responses and phytohormone crosstalk. Front. Plant Sci. 7: 1884. 10.3389/fpls.2016.01884 28018412 PMC5159420

[B73] Song Y. Xiang F. Zhang G. Miao Y. Miao C. Song C. P. (2016). Abscisic acid as an internal integrator of multiple physiological processes modulates leaf senescence onset in Arabidopsis thaliana. Front. Plant Sci. 7:181. 10.3389/fpls.2016.00181 26925086 PMC4759271

[B74] Strasser R. J. Srivastava A. Tsimilli-Michael M. (2000). “The fluorescence transient as a tool to characterize and screen photosynthetic samples,” in Probing photosynthesis: mechanisms, regulation and adaptation. Eds. Yunus M. Pathre U. Mohanty P. (London: Taylor and Francis), 445–483.

[B75] Strasser R. J. Tsimilli-Michael M. Srivastava A. (2004). “Analysis of the chlorophyll a fluorescence transient,” in Chlorophyll a Fluorescence. Eds. Papageorgiou G. C. Govindjee (Dordrecht: Springer Netherlands), 321–362.

[B76] Su M. Huang G. Zhang Q. Wang X. Li C. Tao Y. (2016). The LEA protein, ABR, is regulated by ABI5 and involved in dark-induced leaf senescence in Arabidopsis thaliana. Plant Sci. 247, 93–103. 10.1016/j.plantsci.2016.03.009 27095403

[B77] Szarejko I. Szurman-Zubrzycka M. Nawrot M. Marzec M. Gruszka D. Kurowska M. (2017). “Creation of a TILLING Population in Barley After Chemical Mutagenesis with Sodium Azide and MNU,” in Biotechnologies for Plant Mutation Breeding. Eds. Jankowicz-Cieslak J. Tai T. H. Kumlehn J. Till B. J. (Switzerland: Springer International Publishing), 91–111. 10.1007/978-3-319-45021-6

[B78] Szurman-Zubrzycka M. E. Zbieszczyk J. Marzec M. Jelonek J. Chmielewska B. Kurowska M. M. (2018). HorTILLUS—A Rich and Renewable Source of Induced Mutations for Forward/Reverse Genetics and Pre-breeding Programs in Barley (*Hordeum vulgare* L.). Front. Plant Sci. 9 216. 10.3389/fpls.2018.00216 29515615 PMC5826354

[B79] Tester M. Bacic A. (2005). Abiotic stress tolerance in grasses. From model plants to crop plants. Plant Physiol. 137, 791–793. 10.1104/pp.104.900138 15761207 PMC1065378

[B80] Tezuka K. Taji T. Hayashi T. Sakata Y. (2013). A novel *abi5* allele reveals the importance of the conserved Ala in the C3 domain for regulation of downstream genes and salt tolerance during germination in Arabidopsis. Plant Signac. Behav. 8, e23455. 10.4161/psb.23455 PMC3676515 23299338

[B81] Utsugi S. Ashikawa I. Nakamura S. Shibasaka M. (2020). TaABI5, a wheat homolog of Arabidopsis thaliana ABA insensitive 5, controls seed germination. J. Plant Res. 133, 245–256. 10.1007/s10265-020-01166-3 32048094

[B82] Vinocur B. Altman A. (2005). Recent advances in engineering plant tolerance to abiotic stress: achievements and limitations. Curr. Opin. Biotechnol. 16, 123–132. 10.1016/j.copbio.2005.02.001 15831376

[B83] Wang X. Guo C. Peng J. Li C. Wan F. Zhang S. (2019). ABRE-BINDING FACTORS play a role in the feedback regulation of ABA signaling by mediating rapid ABA induction of ABA co-receptor genes. New Phytol. 221, 341–355. 10.1111/nph.15345 30019753

[B84] Xu Z. Y. Yoo Y. J. Hwang I. (2014). “ABA Conjugates and Their Physiological Roles in Plant Cells,” in Abscisic acid: Metabolism, Transport and Signaling. Ed. Zhang D. (Dordrecht: Springer Netherlands), 77–87.

[B85] Xue G. P. Loveridge C. W. (2004). HvDRF1 is involved in abscisic acid-mediated gene regulation in barley and produces two forms of AP2 transcriptional activators, interacting preferably with a CT-rich element. Plant J. 37, 326–339. 10.1046/j.1365-313X.2003.01963.x 14731254

[B86] Yan F. Deng W. Wang X. Yang C. Li Z. (2012). Maize (*Zea mays* L.) homologue of ABA-insensitive (ABI) 5 gene plays a negative regulatory role in abiotic stresses response. Plant Growth Regul. 68, 383–393. 10.1007/s10725-012-9727-x

[B87] Yang J. Worley E. Udvardi M. (2014). A NAP-AAO3 regulatory module promotes chlorophyll degradation via ABA biosynthesis in Arabidopsis leaves. Plant Cell 26, 4862–4874. 10.1105/tpc.114.133769 25516602 PMC4311216

[B88] Yoshida T. Fujita Y. Sayama H. Kidokoro S. Maruyama K. Mizoi J. (2010). AREB1, AREB2, and ABF3 are master transcription factors that cooperatively regulate ABRE-dependent ABA signaling involved in drought stress tolerance and require ABA for full activation. Plant J. 61, 672–685. 10.1111/j.1365-313X.2009.04092.x 19947981

[B89] Yoshida T. Fujita Y. Maruyama K. Mogami J. Todaka D. Shinozaki K. (2015). Four A rabidopsis AREB/ABF transcription factors function predominantly in gene expression downstream of SnRK2 kinases in abscisic acid signalling in response to osmotic stress. Plant Cell Environ. 38, 35–49. 10.1111/pce.12351 24738645 PMC4302978

[B90] Yoshida T. Christmann A. Yamaguchi-Shinozaki K. Grill E. Fernie A. R. (2019). Revisiting the Basal Role of ABA–Roles Outside of Stress. Trends Plant Sci. 24, 625–635. 10.1016/j.tplants.2019.04.008 31153771

[B91] Yu F. Wu Y. Xie Q. (2015). Precise protein post-translational modifications modulate ABI5 activity. Trends Plant Sci. 20, 569–575. 10.1016/j.tplants.2015.05.004 26044742

[B92] Yuan K. Rashotte A. M. Wysocka-Diller J. W. (2011). ABA and GA signaling pathways interact and regulate seed germination and seedling development under salt stress. Acta Physiol. Plant 33, 261–271. 10.1007/s11738-010-0542-6

[B93] Zhang Y. Sun Q. Zhang C. Hao G. Wang C. Dirk L. M. (2019). Maize VIVIPAROUS1 interacts with ABA INSENSITIVE5 to regulate GALACTINOL SYNTHASE2 expression controlling seed raffinose accumulation. J. Agric. Food Chem. 67, 4214–4223. 10.1021/acs.jafc.9b00322 30915847

[B94] Zheng Y. Schumaker K. S. Guo Y. (2012). Sumoylation of transcription factor MYB30 by the small ubiquitin-like modifier E3 ligase SIZ1 mediates abscisic acid response in Arabidopsis thaliana. Proc. Natl. Acad. Sci. U. S. A. 109, 12822–12827. 10.1073/pnas.1202630109 22814374 PMC3411956

[B95] Zhou X. Hao H. Zhang Y. Bai Y. Zhu W. Qin Y. (2015). SOS2-LIKE PROTEIN KINASE5, an SNF1-RELATED PROTEIN KINASE3-type protein kinase, is important for abscisic acid responses in Arabidopsis through phosphorylation of ABSCISIC ACID-INSENSITIVE5. Plant Physiol. 168, 659–676. 10.1104/pp.114.255455 25858916 PMC4453773

[B96] Zinsmeister J. Lalanne D. Terrasson E. Chatelain E. Vandecasteele C. Vu B. L. (2016). ABI5 is a regulator of seed maturation and longevity in legumes. Plant Cell 28, 2735–2754. 10.1105/tpc.16.00470 27956585 PMC5155344

[B97] Zou M. Guan Y. Ren H. Zhang F. Chen F. (2008). A bZIP transcription factor, OsABI5, is involved in rice fertility and stress tolerance. Plant Mol. Biol. 66, 675–683. 10.1007/s11103-008-9298-4 18236009

