# Supplementary material for: Barley ABI5 (Abscisic Acid INSENSITIVE 5) Is Involved in Abscisic Acid-Dependent Drought Response
Source: Front Plant Sci. 2020 Jul 29;11:1138. doi: 10.3389/fpls.2020.01138 (PMC7405899; doi:10.3389/fpls.2020.01138)
Supplement: Supplementary file 1 [file DataSheet_1.docx]

**Supplementary Material S1**: Composition of nutrient solution used in drought experiment. Each box was supplemented with 90 ml of mineral compound mixture and 10 ml of additional nitrate solution.

| **Mixture of mineral compounds** | |
| --- | --- |
| **Compound** | **Weight per 1 L (g)** |
| NH_4_NO_3_ | 34.3 |
| KH_2_PO_4_ | 40.8 |
| K_2_SO_4_ | 10 |
| MgSO_4_×7H_2_O | 61.5 |
| H_3_BO_3_ | 0.05 |
| CuSO_4_ | 0.02 |
| MnSO_4_×H_2_O | 0.01 |
| Fe(C_6_H_5_O_7_)×3H_2_O | 0.5 |

**Additional nitrate solution**

3.43 g of NH_4_NO_3_ was dissolved in 10 ml of water.
