# Supplementary material for: Barley ABI5 (Abscisic Acid INSENSITIVE 5) Is Involved in Abscisic Acid-Dependent Drought Response
Source: Front Plant Sci. 2020 Jul 29;11:1138. doi: 10.3389/fpls.2020.01138 (PMC7405899; doi:10.3389/fpls.2020.01138)

**Supplementary Material S2**: The schedule of drought experiment with indicated assays conducted on 10, 13, 15 and 25 DAS.


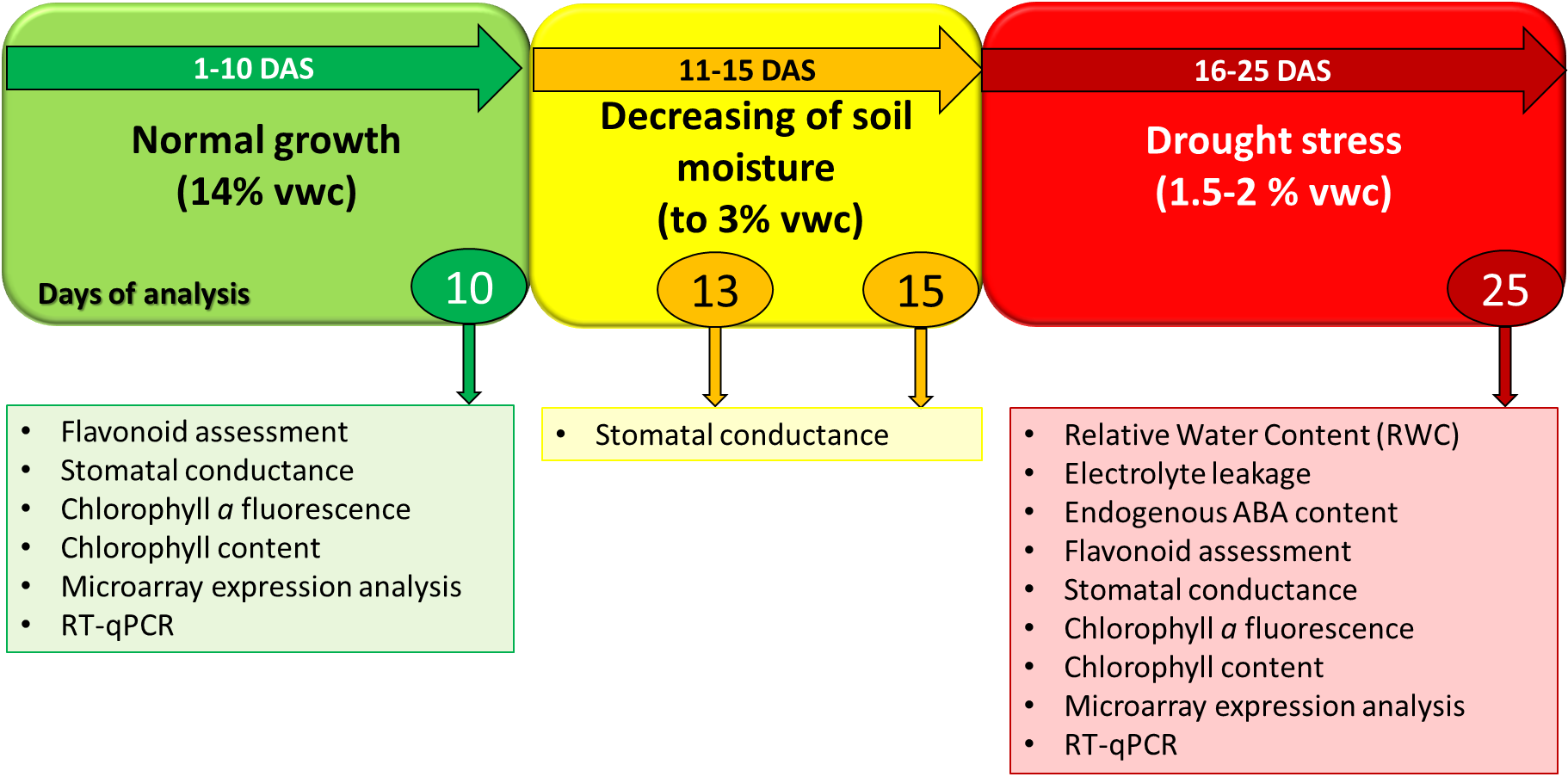

Supplement: Supplementary file 2 [file DataSheet_2.docx]
