# Supplementary material for: Barley ABI5 (Abscisic Acid INSENSITIVE 5) Is Involved in Abscisic Acid-Dependent Drought Response
Source: Front Plant Sci. 2020 Jul 29;11:1138. doi: 10.3389/fpls.2020.01138 (PMC7405899; doi:10.3389/fpls.2020.01138)
Supplement: Supplementary file 3 [file DataSheet_3.docx]

**Supplementary Material S3**: The sequences of primers used for RT-qPCR analysis of ABA-related genes

| **Gene** | **Primer forward** | **Primer reverse** |
| --- | --- | --- |
| ***HvGAPDH*** | TGCTGCCAAGGCTGTTGGTAAG | AGTGGGAACCCGGAAAGACATAC |
| ***HvEF1*** | CCCTCCTCTTGGTCGTTTTG | ATGACACCAACAGCCACAGTTT |
| ***HvABI5*** | CCGGTCCCTGTTGCCCCTAAAG | CGCCGCCCATACCGAG |
| ***HVA1*** | GGCGAGACGGTGGTGAAC | AGTGATTCCTGGTGGTGGTG |
| ***HVA22*** | TGTGGTACCCAGTGAAGCTG | CCTGAGCTGCTCCCTGAC |
| ***HvDRF1*** | GTGGCAACCGAAGGTGTAGT | AGCAAATCTGACCGAGAGGA |
| ***HvNCED1*** | CCAGCACTAATCGATTCC | GAGAGTGGTGATGAGTAA |
| ***HvAO5b*** | TTGGCGTTGTGATTGCTGAGAC | AAAACGGGGGAGGATGGAAGTA |
| ***HvBG4*** | CCCGCCGGAGTTCGTCTTC | TCCTCAGCCACAGCACCCTCAT |
| ***HvBG8*** | CCCCGGCCAGGCGTATTCC | TCCCAGGCTTATTCGTCATCCA |
| ***HvPYL5*** | CCGCCGGCAGAATAACGAC | CCCTCCCGAGAAAAAGCAAAGA |
| ***HvSnRK2.1*** | GCAGAAACCGGGCGATAACG | GCTCCCCAGGCAGGCAACC |
| ***HvPP2C4*** | TGGCCTCTGGGATGTATTGTCG | GAGCCGCTGGATCTGGGGAGTC |
