# Supplementary material for: Barley ABI5 (Abscisic Acid INSENSITIVE 5) Is Involved in Abscisic Acid-Dependent Drought Response
Source: Front Plant Sci. 2020 Jul 29;11:1138. doi: 10.3389/fpls.2020.01138 (PMC7405899; doi:10.3389/fpls.2020.01138)

**Supplementary Material S5**: Alignment of ABI5 and ABF protein sequences across monocot and dicot species using ClustalOmega (<http://www.ebi.ac.uk/Tools/msa/clustalo/>) and Jalview 2.11.0 with marked arginine (R274) changed in *hvabi5.d* (red frame). Mutation positions of alleles for which initial drought experiment was performed (*hvabi5.b*, *hvabi5.e, hvabi5.i, hvabi5.o,* *hvabi5.u* and *hvabi5.w*) are indicated by red arrows. C1, C2, C3 – conserved charged domains, bZIP – basic leucine zipper domain. Blue color indicates conservation of aligned position. Yellow bars – level of conservation and alignment quality, black bars – level of consensus sequence, grey bars – number of aligned positions. Note, that numbering visible in alignment visualization refers to multi sequence alignment of 17 ABI5 and ABF protein sequences in dicot and monocot species. It does not refer to the single HvABI5 protein and does not indicate the position of amino acids substituted in the identified mutants given in Tab. 1.

*hvabi5.o*

**C1**


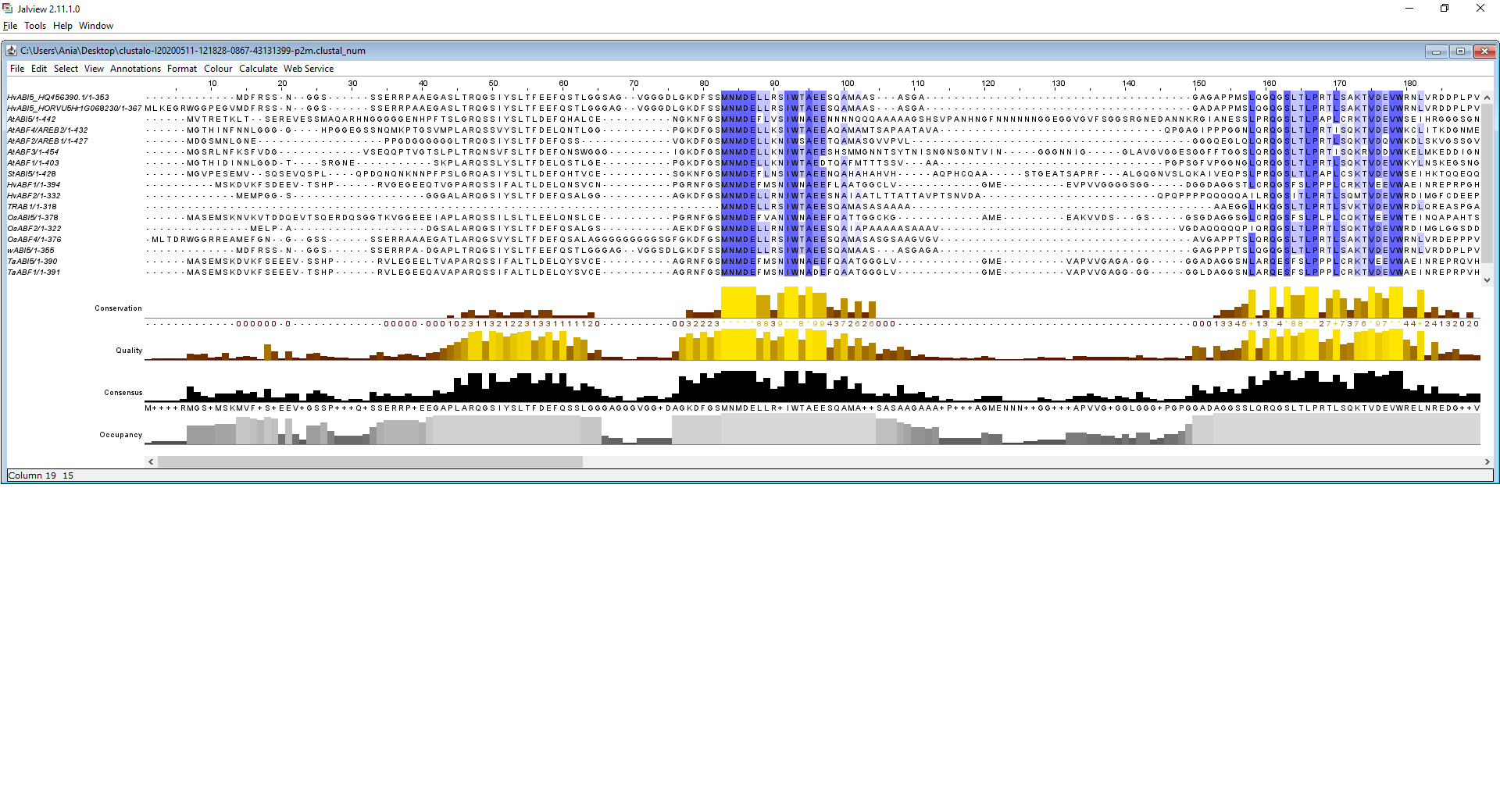


*hvabi5.u*

*hvabi5.i*

**C2**


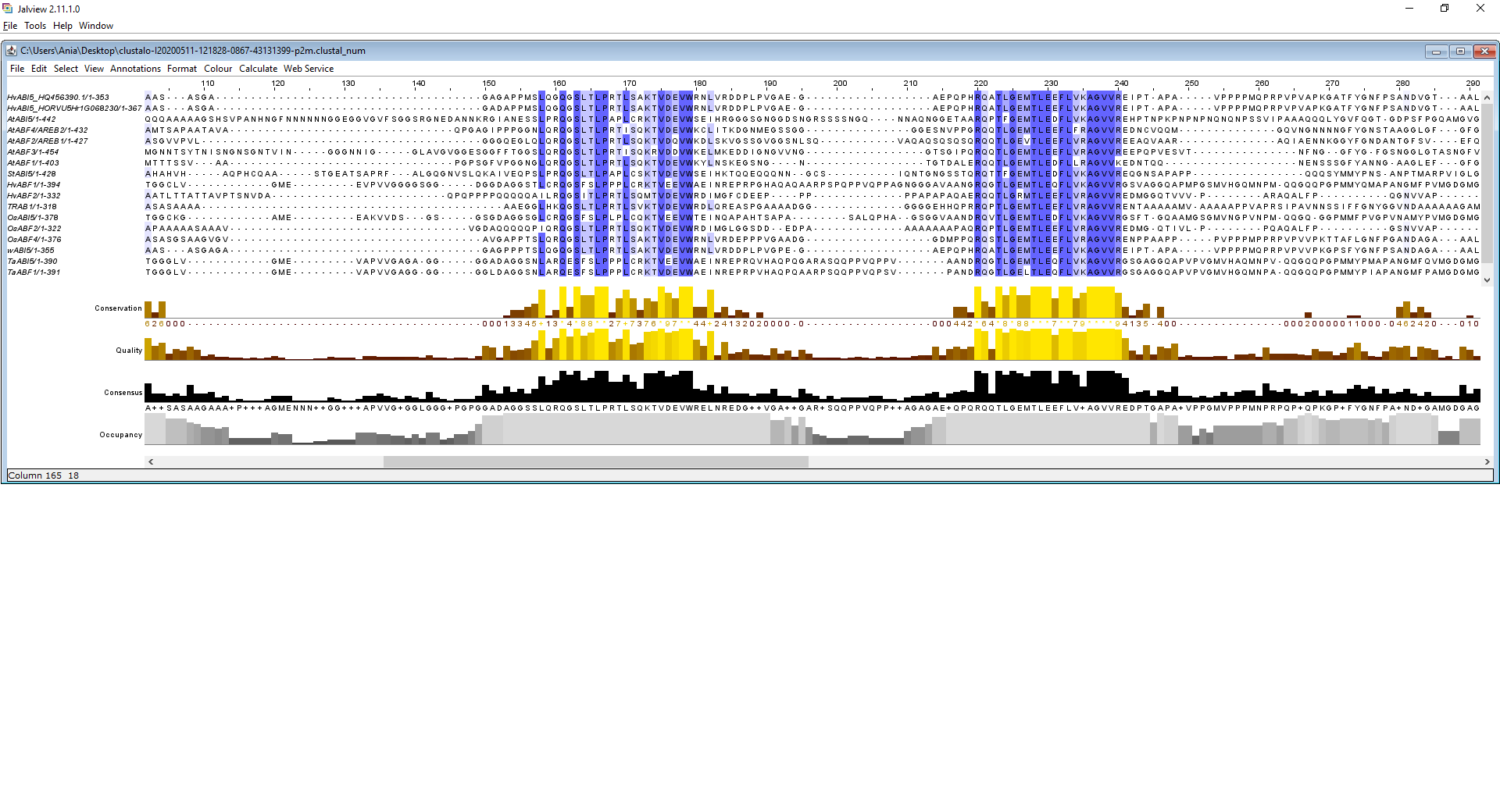


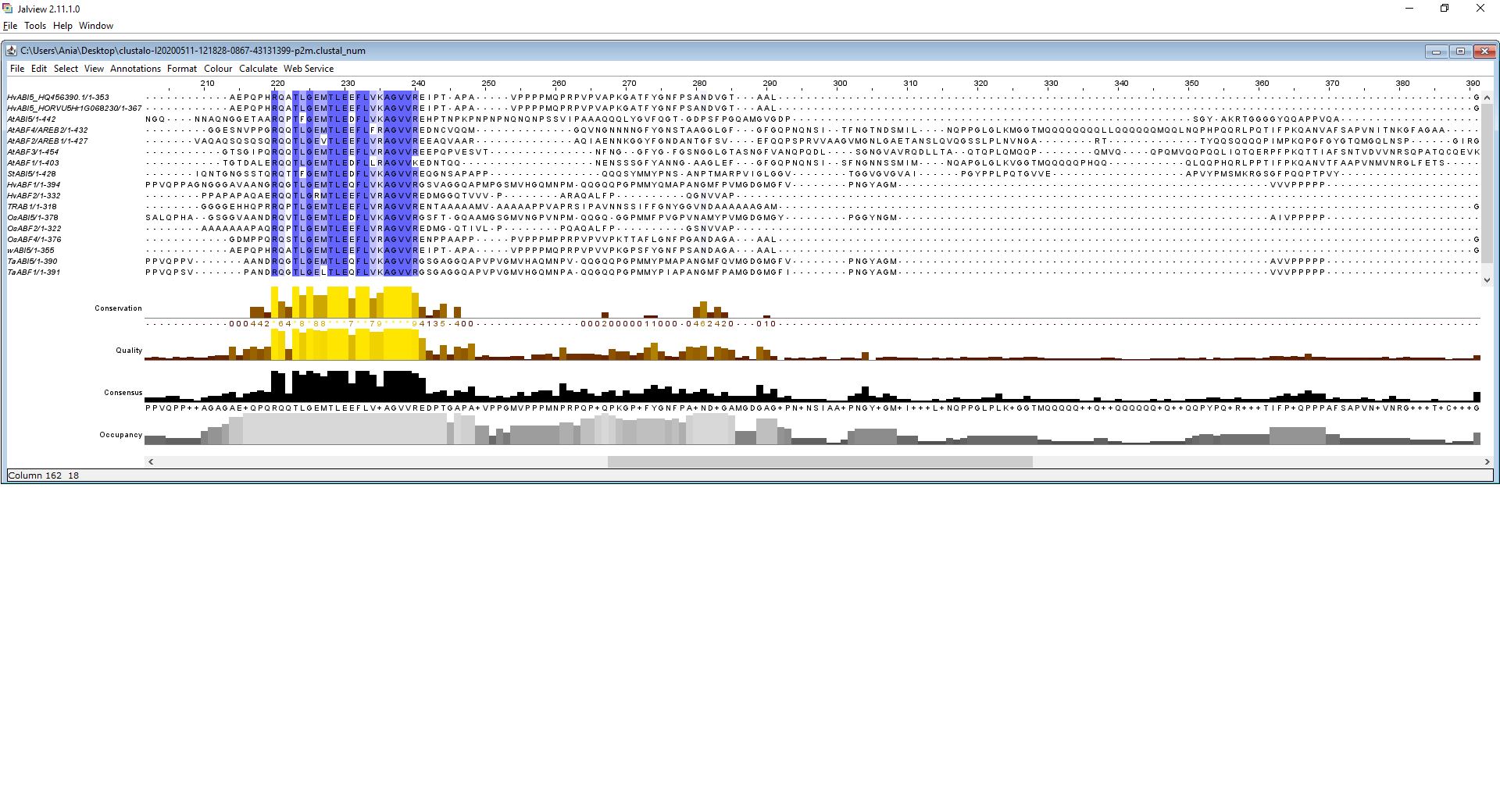


*hvabi5.w*

*hvabi5.e*

**C3**


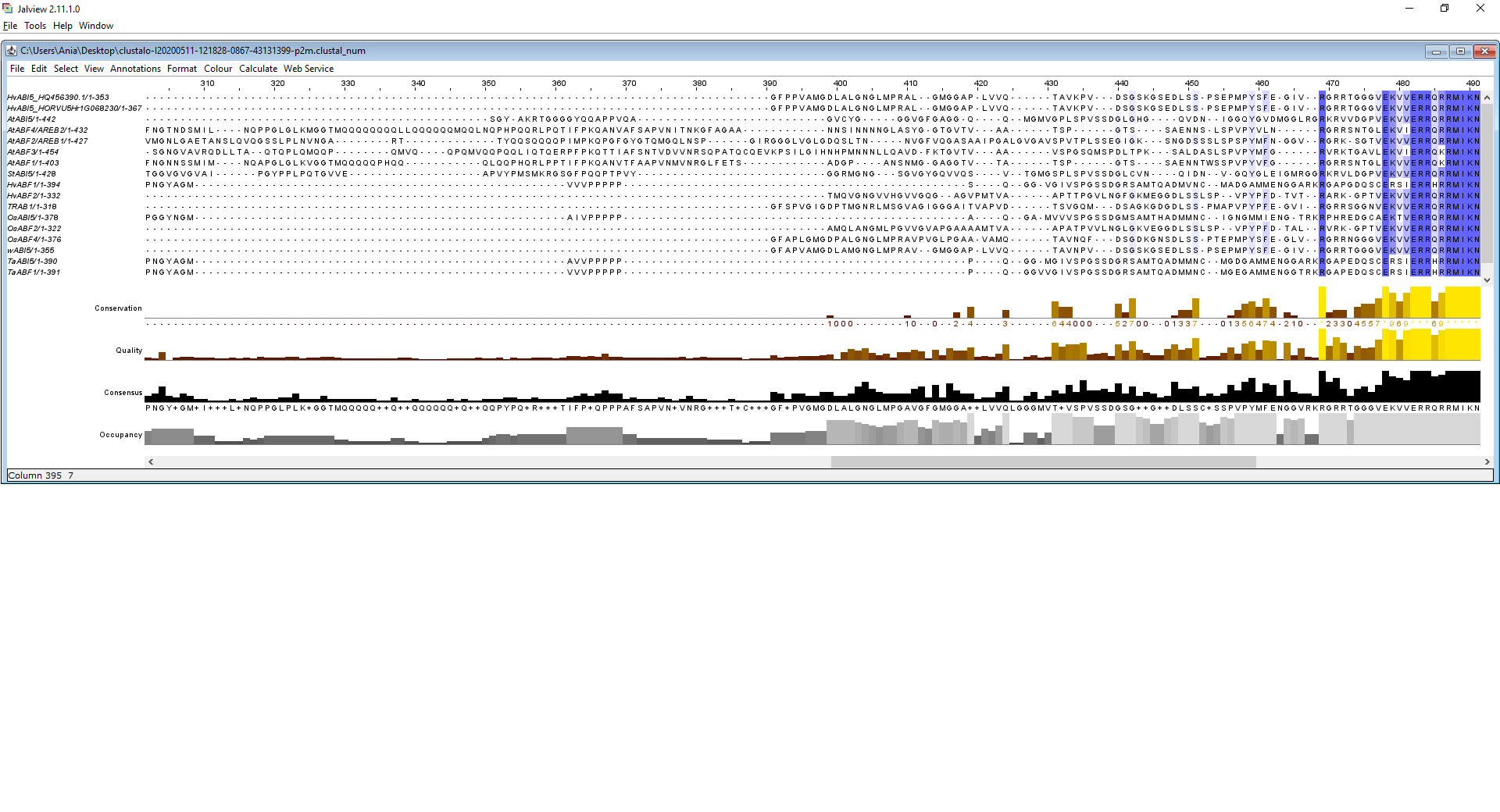


*hvabi5.b*

**bZIP**

R274

***hvabi5.d***


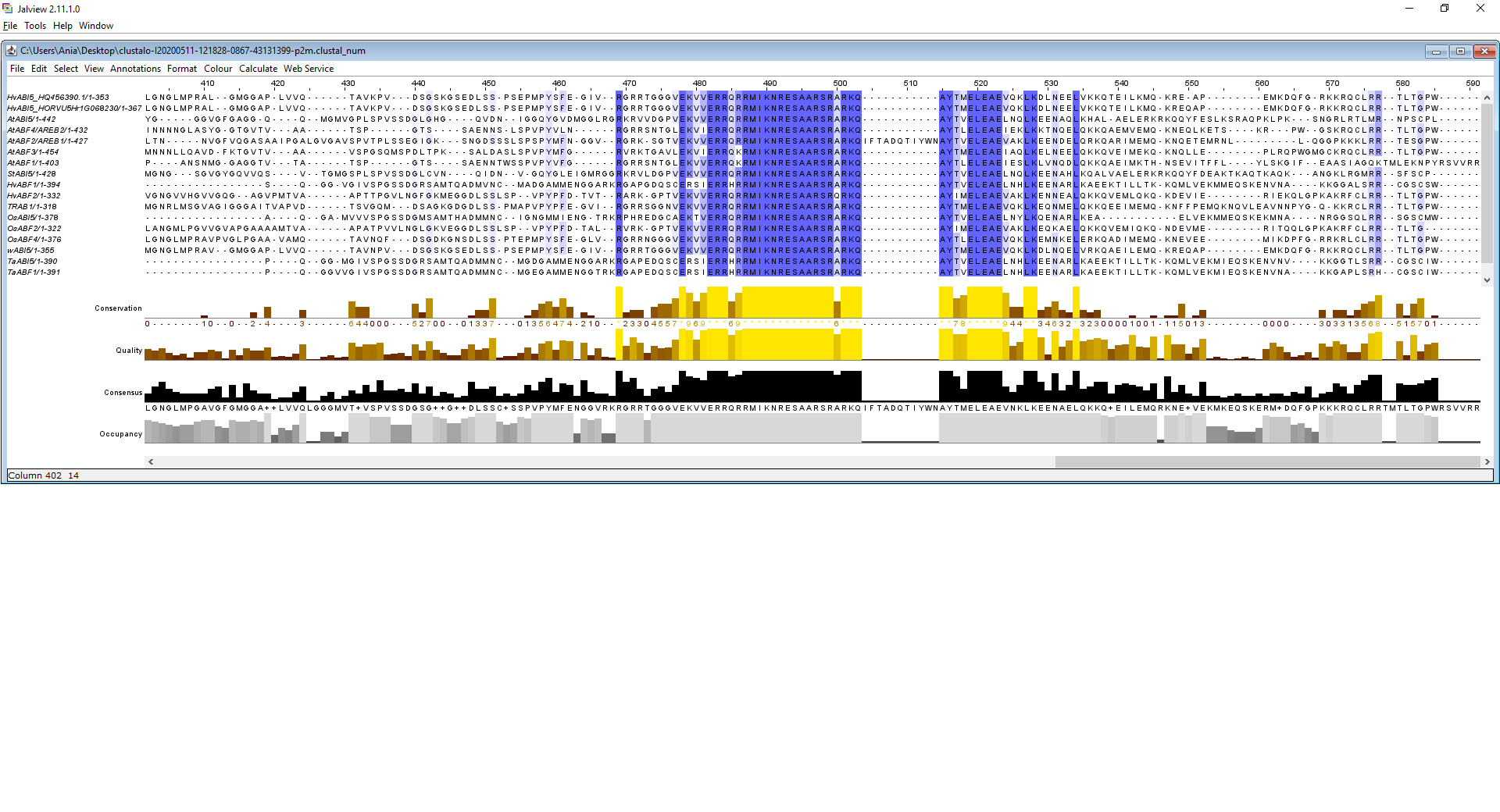


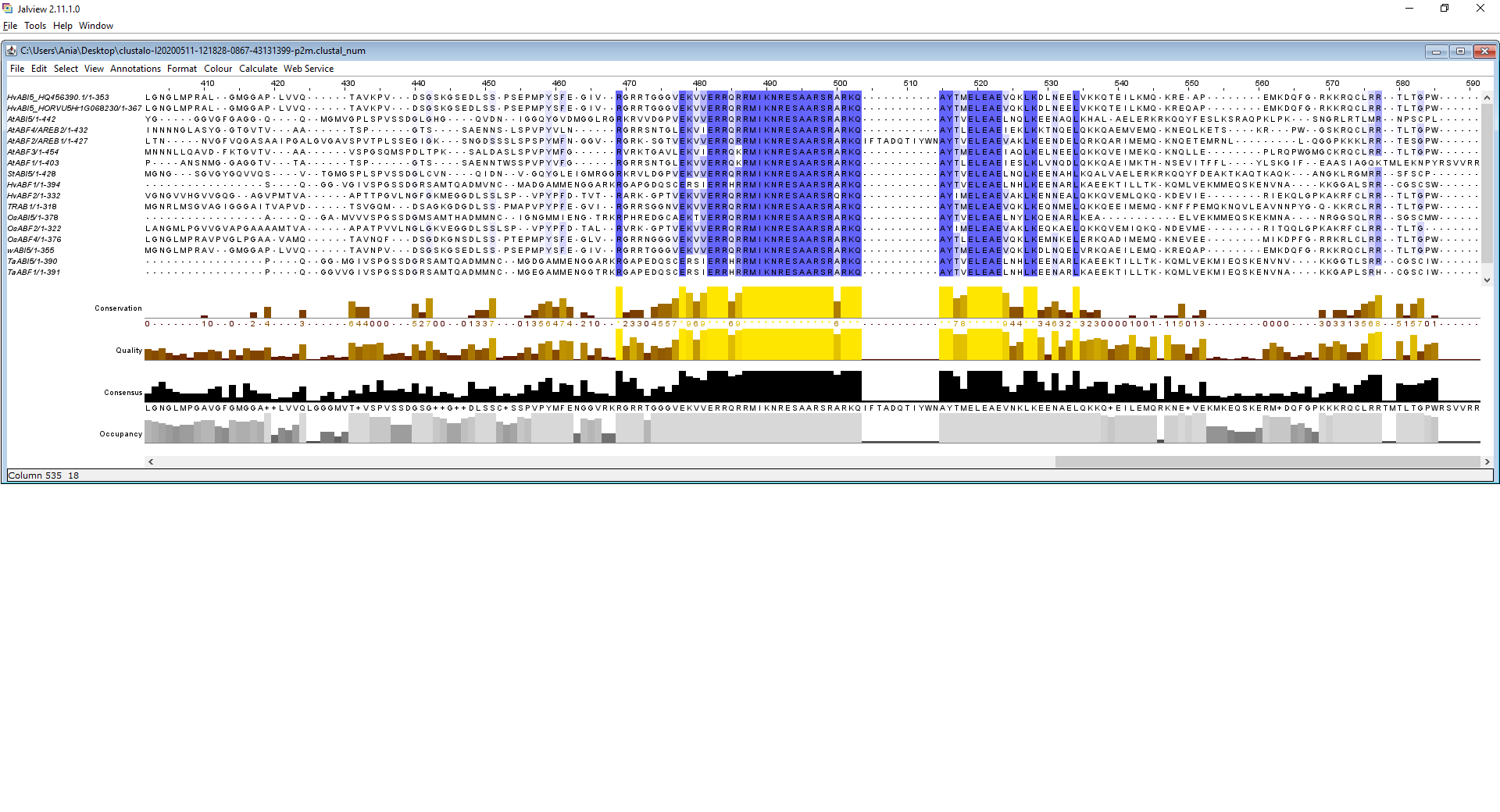

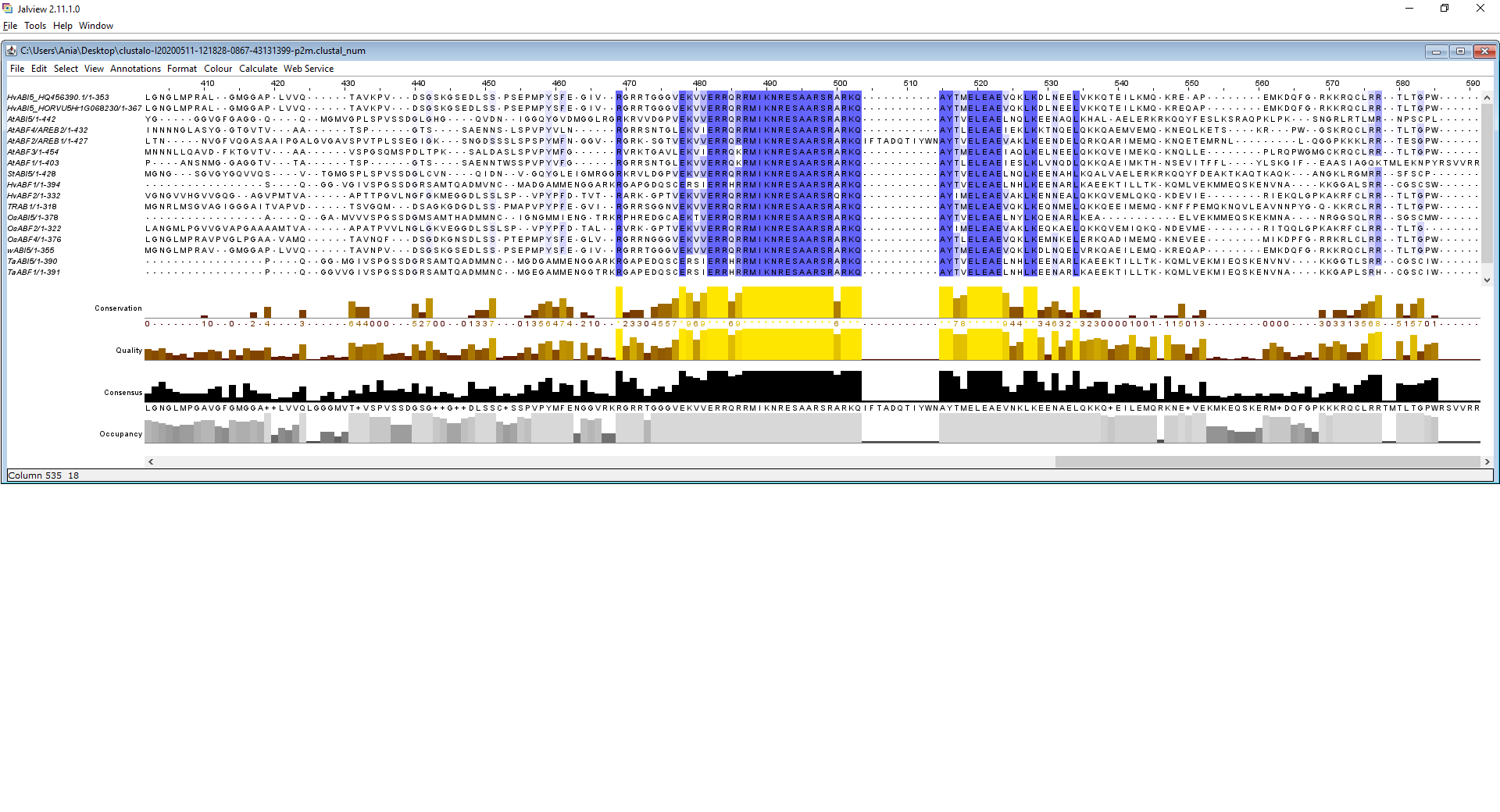

Supplement: Supplementary file 5 [file DataSheet_5.docx]
