# Supplementary material for: Barley ABI5 (Abscisic Acid INSENSITIVE 5) Is Involved in Abscisic Acid-Dependent Drought Response
Source: Front Plant Sci. 2020 Jul 29;11:1138. doi: 10.3389/fpls.2020.01138 (PMC7405899; doi:10.3389/fpls.2020.01138)

**Supplementary Material S6**: The growth of the first leaf of WT and *hvabi5.d* seedlings after 6 days of ABA treatment. The statistical analysis was performed using the two-way ANOVA (P≤0.05) followed by Tukey’s honestly significant difference test (Tukey HSD-test) (P≤0.05) to assess the differences between different growth conditions and genotypes. Statistically significant differences (P≤0.05) are marked by different letters.


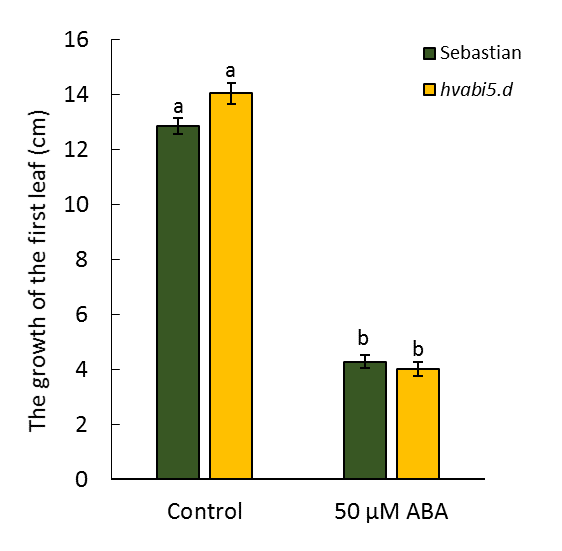

Supplement: Supplementary file 6 [file DataSheet_6.docx]
