# Supplementary material for: Barley ABI5 (Abscisic Acid INSENSITIVE 5) Is Involved in Abscisic Acid-Dependent Drought Response
Source: Front Plant Sci. 2020 Jul 29;11:1138. doi: 10.3389/fpls.2020.01138 (PMC7405899; doi:10.3389/fpls.2020.01138)

**Supplementary Material S7**: Photosynthesis parameters in ‘Sebastian’ and *hvabi5.d* based on chlorophyll *a* fluorescence in the presence of drought (25 DAS). The statistical analysis was calculated using two-way ANOVA (P≤0.05) followed by Tukey’s honestly significant difference test (Tukey HSD-test) (P≤0.05) to assess differences between growth conditions and analyzed genotypes. Statistically significant differences (P≤0.05) are marked by different letters. ABS/RC - absorption flux per active reaction center, TR_0_/RC - trapping flux per RC, ET_0_/RC - electron-transport flux per RC, DI_0_/RC - dissipation flux per RC, RC per cross section RC/CS_0_, φP_0_ - maximum quantum yield of primary photochemistry, PI_ABS_ - performance index.


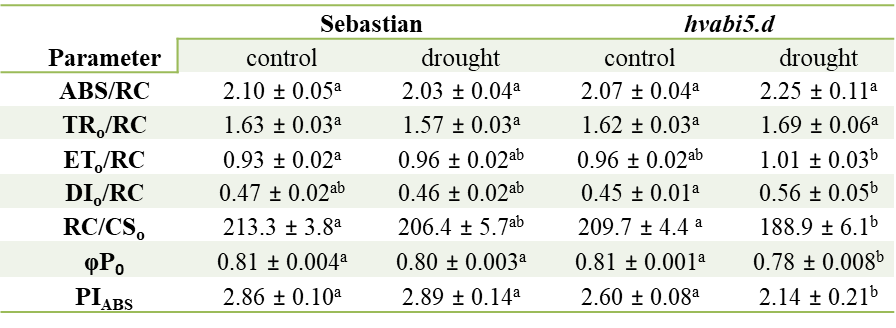

Supplement: Supplementary file 7 [file DataSheet_7.docx]
