# Supplementary material for: Barley ABI5 (Abscisic Acid INSENSITIVE 5) Is Involved in Abscisic Acid-Dependent Drought Response
Source: Front Plant Sci. 2020 Jul 29;11:1138. doi: 10.3389/fpls.2020.01138 (PMC7405899; doi:10.3389/fpls.2020.01138)

**Supplementary Material S8**: Validation of Agilent Barley Gene Expression Arrays by RT-qPCR of selected DEGs. **(A)** The relative expression of selected genes and **(B)** the correlation between data obtained from Agilent Barley Gene Expression Arrays and qRT-PCR analyses.


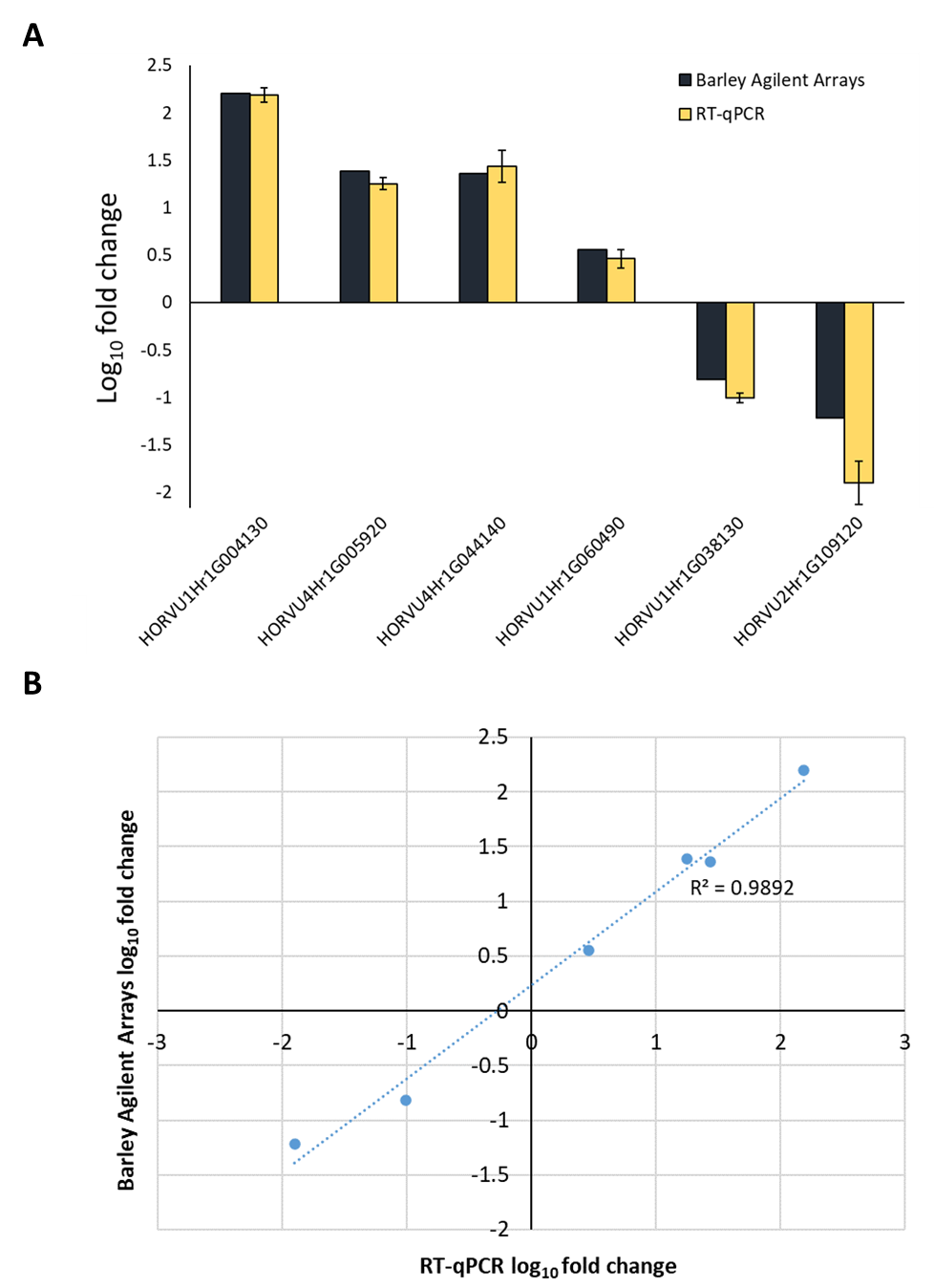

Supplement: Supplementary file 8 [file DataSheet_8.docx]
