# Supplementary material for: Barley ABI5 (Abscisic Acid INSENSITIVE 5) Is Involved in Abscisic Acid-Dependent Drought Response
Source: Front Plant Sci. 2020 Jul 29;11:1138. doi: 10.3389/fpls.2020.01138 (PMC7405899; doi:10.3389/fpls.2020.01138)
Supplement: Supplementary file 12 [file DataSheet_12.docx]

**Supplementary Material S12.** Putative motifs for AtABI5 binding in the promoters (-1000 bp from ATG START codon) of *HvNCED1*, *HvSnRK2.1* and *HvPP2C4*.

| **Potential target gene** | **Position** | **Strand** | **Motif recognized by AtABI5** |
| --- | --- | --- | --- |
| *HvNCED1*  (HORVU5Hr1G008050) | -479 to -461 | + | 5’TGAACC**ACGTG**GTCTCCT3’ |
|  | -461 to -479 | - | 5’AGGAGACC**ACGTG**GTTCA3’ |
| *HvPP2C4*  (HORVU3Hr1G050340) | -954 to -936 | + | 5’GGCTGATG**ACATGGC**ATA3’ |
|  | -843 to -823 | + | 5’GGAGGGAC**ACGTG**TGTGA3’ |
|  | -823 to 843 | - | 5’TCACAC**ACGTG**TCCCTCC3’ |
|  | -567 to -585 | - | 5’CCGGGGACG**CGTGTC**GAG3’ |
| *HvSnRK2.1*  (HORVU2Hr1G110230) | -194 to -212 | - | 5’CGTCGGCC**ATGTGTC**GCC3’ |
