# Supplementary material for: Barley ABI5 (Abscisic Acid INSENSITIVE 5) Is Involved in Abscisic Acid-Dependent Drought Response
Source: Front Plant Sci. 2020 Jul 29;11:1138. doi: 10.3389/fpls.2020.01138 (PMC7405899; doi:10.3389/fpls.2020.01138)

**Supplementary Material S13**: Response of *hvabi5.d* F_4_BC_2_ and ‘Sebastian’ to drought stress **(A)** Relative Water Content on 25 DAS under optimal water conditions and drought in *hvabi5.d* F4BC2 and ‘Sebastian’. The statistical significance was estimated by T-test to assess the differences between genotypes - *P≤0.05, **P≤0.01, ***P≤0.001. **(B)** The flavonol content and **(C)** the anthocyanin content before and after drought, on 10 and 25 DAS in *hvabi5.d* F4BC2 and ‘Sebastian’ **(D)** the stomatal conductance (g_s_) on the 10, 13, 15 and 25 DAS in the analyzed genotypes. The statistical analysis was performed using the two-way ANOVA (P ≤ 0.05) followed by Tukey’s honestly significant difference test (Tukey HSD-test) (P ≤ 0.05) to assess the differences between different growth conditions and between genotypes. Statistically significant differences (P ≤ 0.05) are marked by different letters. **(E)** The expression of *HvABI5*-related genes and ABA pathway-related genes in *hvabi5.d* F_4_BC_2_ and its WT parent ‘Sebastian’ under optimal water supply (10 DAS) and drought stress (25 DAS). *HvDRF1 - DEHYDRATION‐RESPONSIVE FACTOR 1*, *HvNCED1* - *CAROTENOID CLEAVAGE DIOXYGENASE1, HvBG* - *β-GLUSIDASE* and *HvSnRK2.1* - *SNF1-RELATED PROTEIN KINASE 2.1* and *HvPP2C4* – *PROTEIN PHOSPATASE 2C 4*. The statistical significance was estimated by T-test between analyzed genotypes - *P≤0.05, **P≤0.01, ***P≤0.001 and between analyzed treatments - #P≤0.05, ##P≤0.01, ###P≤0.001.


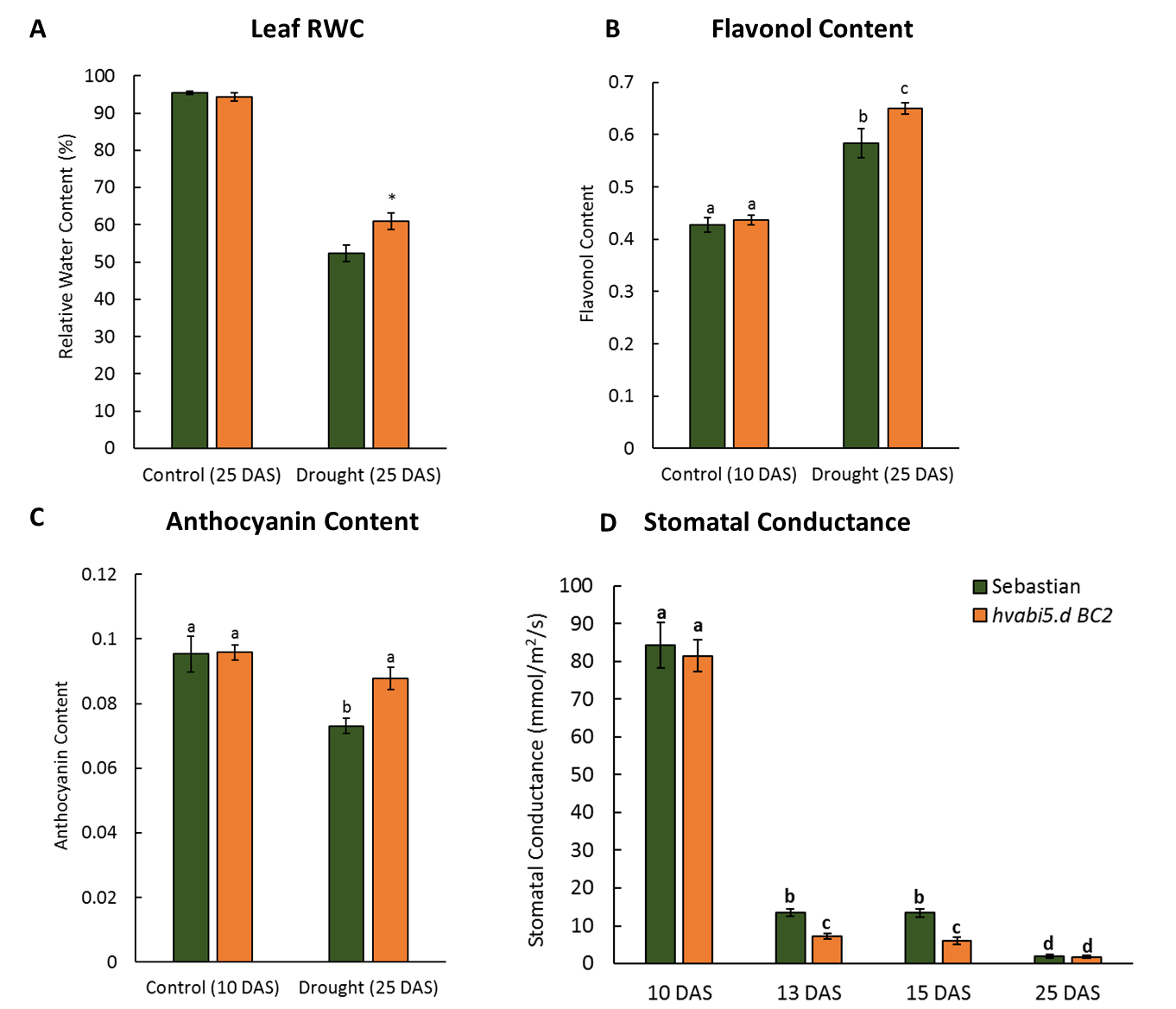


**(E)**


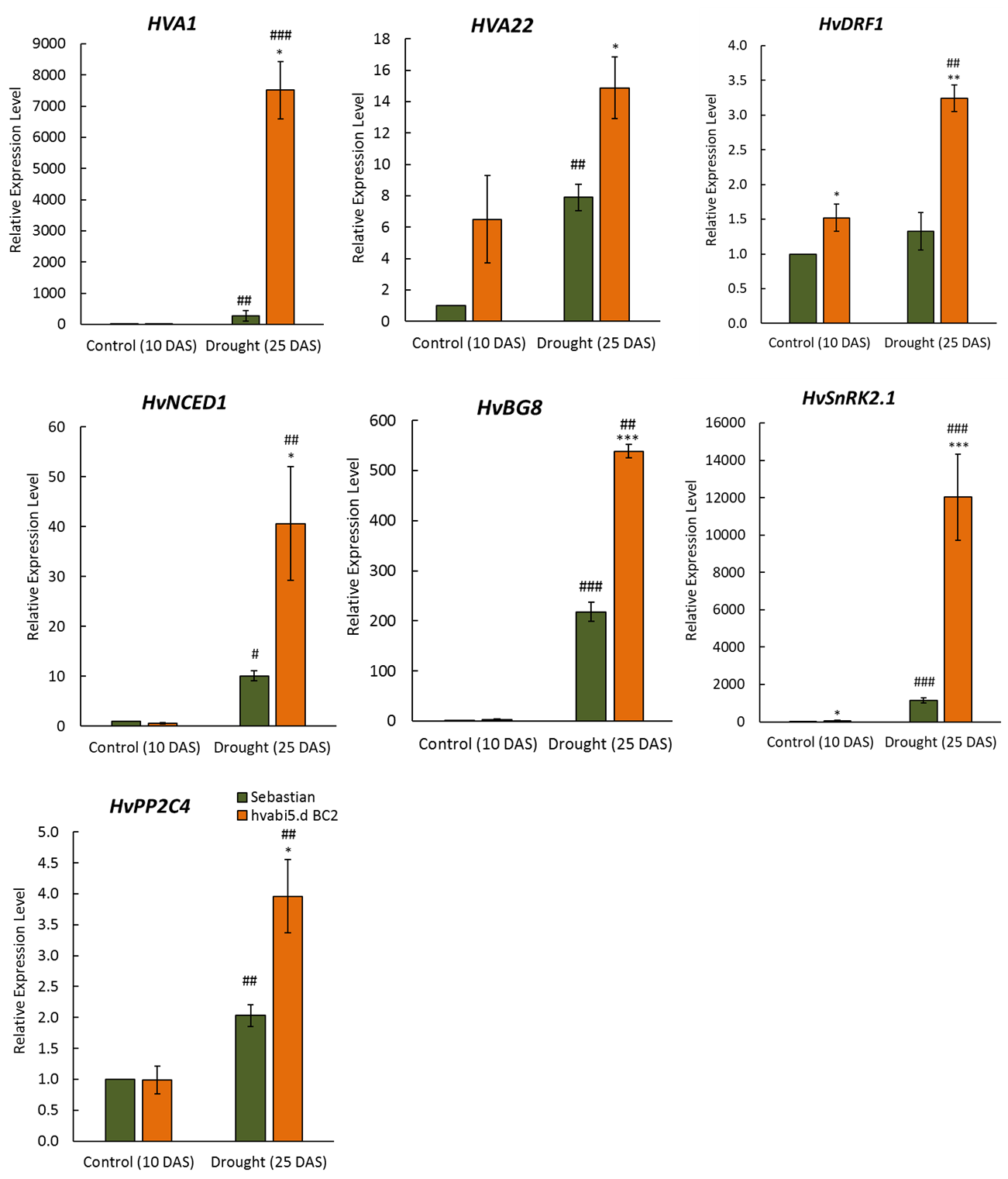

Supplement: Supplementary file 13 [file DataSheet_13.docx]
